# High genus surface parameterization using the Euclidean Ricci flow method

**DOI:** 10.1038/s41598-025-97421-5

**Published:** 2025-05-22

**Authors:** Yuan-guang Wang

**Affiliations:** Beijing Electro-Mechanical Engineering Institute, Beijing, China

**Keywords:** The Euclidean Ricci flow, Surface parameterization, High genus surface, Conformal parameterization, Applied mathematics, Computational science, Computer science

## Abstract

The parameterization of surfaces, which is related to many frontier problems in mathematics, has long been a challenge for scientists and engineers. On the other hand, Ricci flow is a powerful tool in geometric analysis for studying low-dimensional topology. Owing to the natural cooperative impetus, Ricci flow has been increasingly employed to parameterize closed surfaces. However, due to the lack of choices when addressing high genus surfaces, engineers must still rely on the mainstream tool of hyperbolic Ricci flow, which is inconsistent with human intuition. Therefore, this disadvantage is a potential barrier for humans in designing textures in the parameter domain. By making a small modification to traditional Euclidean Ricci flow to sacrifice its tessellation capability, we develop a new Euclidean Ricci flow method with special features characterized by its ability to embed the fundamental domain of high genus surfaces in 2-dimensional Euclidean space. Based on this method, the parameter domain is more suitable for exploring the nature of singularity points on high genus surfaces and more suitable for designing the checkerboard textures. Four illustrative examples demonstrated the robust, rigorous features of our method, abandoning dogma and challenging the traditional views that only the hyperbolic Ricci flow can be used to parameterize high genus surfaces.

## Introduction

### Background

In computer graphics, surface parameterization refers to the process of mapping a surface onto 2D domains. The parameterization of surfaces is a foundational problem in computer graphics and geometry processing and is relevant to many other processes, such as texture mapping, hexahedral mesh generation^1^, surface registration and shape correspondence. With the development of the game and film industry, surface parameterization has become increasingly important for a wide variety of applications ranging from computer graphics to computer vision. Recently, the number of new medical imaging applications has increased.

On the other hand, Ricci flow is a powerful tool in geometric analysis for studying low-dimensional topology. Ricci flow was originally introduced by Richard Hamilton in 1982^2^, and was later used to prove the Poincaré conjecture on a 3D manifold^3^. Marked by the introduction of surface Ricci flow^4^, Ricci flow has started to impact practical fields and address fundamental engineering problems. There is a natural cooperative impetus or drive to involve surface Ricci flow in surface parameterization since surface Ricci flow has unique characteristics, making it outstanding among many surface parameterization methods. First, surface Ricci flow is conformal, namely, the deformation of the Riemannian metric preserves angles. This fact greatly simplifies both theoretical arguments and algorithmic designs. Second, surface Ricci flow seldom blows up; that is, the discrete Gaussian curvature during the flow is always bounded under a certain value. This phenomenon ensures the numerical stability of the discrete surface Ricci flow. Third, surface Ricci flow has intuitive geometric interpretations, directly leading to the design of data structures. Therefore, surface Ricci flow can be used to compute general diffeomorphisms between surfaces. Moreover, surface Ricci flow has demonstrated its great potential by solving various problems in many fields, which can hardly be handled by substitute methods. With the completion of all the theoretical and arithmetic procedures^5^, an increasing number of engineering fields are employing surface Ricci flow to parameterize surfaces^6,7^.

For the niche branch of high genus surface (genus >= 2), there is a consensus that there is only one type of Riemannian metric—a hyperbolic metric on the surface^8,9^. Therefore, when turning to the Ricci flow, researchers simply choose the hyperbolic Ricci flow to achieve the parameterization process^10–13^.

### Motivation and choice of technique route

Although the hyperbolic Ricci flow has been widely employed to parameterize high genus surfaces, its disadvantage is still remarkable: the property of the hyperbolic space represented by the Poincaré disk regarding where to embed the fundamental domain obtained by the hyperbolic Ricci flow is not consistent with human intuition. This fact will dramatically prevent leveraging human intuition to better understand the nature of singularity points on high genus surfaces.

To address this dilemma, a valuable question to consider is whether we can sacrifice the capability to tessellate the fundamental domain to the hyperbolic space to gain other advantages.

According to the famous painting of Escher’s Angels and Devils^14^, the tessellation on the hyperbolic Poincaré disk is truly beautiful but difficult to master for our intuition. This time, we want to temporarily abandon hyperbolic spaces and find alternative ways to parameterize high genus surfaces. As we rethink this issue, we have many reasons to believe it is time to reunite with our old friend—Euclidean space.

Therefore, we attempt to use the Euclidean Ricci flow for extremal length, which is a small modification to the traditional Euclidean Ricci flow^15^ because of the different settings of the target surface curvature to parameterize high genus surfaces. After obtaining the Euclidean metric on the high genus surface, we embed it in the 2-dimensional Euclidean space ${\mathbb{E}}^{2}.$

## Theoretical aspects and algorithms

### Computational conformal geometry

A conformal structure is a feature of angle preservation^9^. Existing in many physics phenomena, ranging from electromagnetic fields to heat diffusion, a conformal structure is a natural geometric structure situated on surfaces such that angles among tangent vectors can be coherently defined on different local coordinate systems. Conformal geometry is a branch of pure mathematics that focuses on conformal structure^8^.

However, computational conformal geometry focuses its attention on the algorithmic issue of conformal geometry and provides useful tools for addressing a broad range of geometric problems in engineering fields. It builds a bridge to link modern pure mathematical theories with real engineering applications.

In computational conformal geometry, a large portion of the focus has been given to discrete surfaces. Therefore, it is necessary for us to transfer the major concept from a smooth surface to a discrete situation. The concepts include (1) the discrete Riemannian metric and (2) the discrete Gaussian curvature.

1. The discrete Riemannian metric on a mesh with Euclidean background geometry is a Euclidean metric with cone singularities. Each vertex is a cone singularity. Similarly, a metric on a mesh with spherical background geometry is a spherical metric with cone singularities, and a metric on a mesh with hyperbolic background geometry is a hyperbolic metric with cone singularities^15^.

The edge length of mesh is suitable to define the discrete Riemannian metric: for every triangle [v_i_, v_j_, v_k_], the triangle inequality holds:

1 $$l([v_{i} ,v_{j} ]) + l([v_{j} ,v_{k} ]) > l([v_{k} ,v_{i} ]).$$

(2) The discrete Gaussian curvature can be calculated from the angle deficit^15^:

2 $$K_{i} = \left\{ {\begin{array}{*{20}c} {2\pi - \sum\limits_{{f_{ijk} \in F}} {\theta_{i}^{jk} } ,v_{i} \notin \partial \Sigma } \\ {\pi - \sum\limits_{{f_{ijk} \in F}} {\theta_{i}^{jk} } ,v_{i} \in \partial \Sigma } \\ \end{array} } \right.,$$

where $\theta_{i}^{jk}$ represents the corner angle attached to vertex v_i_ for the triangle face f_ijk_.$\Sigma$ and $\partial \Sigma$ are the zone and the boundary of the zone, respectively.

### Discrete surface Ricci flow

Among the major branches in computational conformal geometry, the Ricci flow is a prominent and promising direction. Currently, discrete Ricci flow has been applied to compute the uniformization metrics and to design the Gaussian curvature of the target surface. It has been used for surface registration, surface matching, shape classification, shape analysis and many fundamental applications in practice^16^.

#### Discrete conformal deformation

Conformal mappings have a special property since they can turn every infinitesimal circle on the original surface to an infinitesimal circle on the parameter domain and maintain the intersection angles among the infinitesimal circles^8^.

Adopting the pattern of circle packing originally described by Thurston^17^, circles on the texture are mapped to circles on the surface, and their tangency relations are well preserved during the conformal deformation of the Riemannian metric. Chow and Luo established the intrinsic connection between circle packing and surface Ricci flow^5^.

Let Γ be a function defined on vertex to satisfy ${\Gamma }:{\text{V}} \to {\mathbb{R}}$^+^, attaching a radius to the vertex v_i_. Similarly, let Φ be a function defined on the edges ${\Phi }:{\text{E}} \to \left[ {0,{\uppi }/2} \right]$, attaching an acute angle Φ(e_ij_) to every edge e_ij_ and is called a weighted function on the edges. The pair of vertex radius function and edge weight function on a triangular mesh M is called the circle packing metric of M. Finally, we attach a radius γ to every vertex. The two adjacent circles c(v_i_, γ_i_) and c(v_j_, γ_j_) may intersect or be tangent to each other. Two circle packing metrics (M, Γ_1_, Φ_1_) and (M, Γ_2_, Φ_2_) on the same mesh are conformally equivalent if Φ_1_ ≡ Φ_2_. That is to say, a conformal deformation of a circle packing metric only modifies the vertex radii and preserves the intersection angles on the edges.

Furthermore, we define discrete conformal factor on each vertex for a given mesh M. It is a function ${\text{u}}:{\text{V}} \to {\mathbb{R}},$

$${\text{u}}_{{\text{i}}} = {{log~\gamma }}_{{\text{i}}} {\text{.}}$$

For more information on the discrete conformal deformation of surface, please refer to the literature ^15,18,19^.

#### Euclidean discrete surface Ricci flow

Suppose S is a smooth surface with Riemannian metric **g** = **g**_ij_. Ricci flow transforms the metric g(t) according to its induced Gaussian curvature K(t), where t is the time

3 $$\frac{{dg_{ij} (t)}}{dt} = - 2K(t)g_{ij} (t),$$

with a constraint to preserve the total surface area. If we represent the Riemannian metric in the following form^9^:

4 $$g(t) = e^{2u(t)} g(0),$$

the surface Ricci flow can be written as:

5 $$\frac{du(t)}{{dt}} = - K(t).$$

The Ricci flow can be easily modified to compute a metric with a prescribed curvature $\overline{K}$. And the Ricci flow will become

6 $$\frac{du(t)}{{dt}} = 2(\overline{K} - K)g_{ij} (t),$$

with the area preserved constraint.

Given a mesh M with a circle packing metric (M, Γ, Φ), the discrete Ricci flow can be written as:

7 $$\frac{du(t)}{{dt}} = (\overline{K}_{i} - K_{i} (t)),$$

where K_i_(t) and $\overline{K}_{i}$ are the current Gaussian curvature and target Gaussian curvature of vertex v_i_, respectively.

In Euclidean discrete surface Ricci flow, the surface is assumed to be located in ${\mathbb{E}}^{3}$ and approximated by a piecewise-linear triangle mesh. Each face on the mesh is a Euclidean triangle. The corner angles and edge lengths are governed by the Euclidean cosine law. In this case, the mesh has Euclidean background geometry.

The calculation results of the traditional Euclidean discrete surface Ricci flow can be found in literature^15^, which focused only on the genus 1 surface.

Essentially, the Euclidean surface Ricci flow applied in this paper is called the Euclidean discrete surface Ricci flow for extremal length, which partly arises from the idea of Lars V. Ahlfors^20,21^. In addition, the only difference between our method and Jin’s traditional Euclidean Ricci flow^15^ is the setting of the target curvature for the parameter domain. In the latter method, the target curvature for all the points will be set to zero^9^, while the setting in our method will be later explained in subsection  “Pushing forward the triangular meshes to the parameter domain for genus 2”. The different settings lead to different appearances after embedding: the embedded zone for the latter case is an irregular 2D shape^15^, whereas the embedded zone in our method will take the form of a combination of multiple rectangles (shown in Fig. 4 and Fig. 26 for genus 2 and genus 3, respectively).

In addition, the rest of this paper will only discuss our method without the involvement of Jin’s method^15^. For conciseness, we refer to our Euclidean discrete surface Ricci flow for extremal length as Euclidean Ricci flow hereinafter unless otherwise specified.

#### Hyperbolic discrete surface Ricci flow

In hyperbolic discrete surface Ricci flow, the surface is assumed to be embedded in the hyperbolic background geometry. Each face on the mesh is a hyperbolic triangle. The corner angles and the edge lengths are governed by the hyperbolic cosine law.

The cosine laws for triangles in hyperbolic geometry is written as:

8 $$\cosh l_{k}^{2} = \cosh l_{i} \cosh l_{j} + \sinh l_{i} \sinh l_{j} \cos \theta_{k} .$$

The discrete Ricci flow for hyperbolic geometry has the same form with Eq. (7).

For more details on the hyperbolic Ricci flow, we refer readers to the comprehensive survey^16,18^.

## Illustrative examples with technical details

In this section, we choose to use the Euclidean Ricci flow to finish the parameterization of high genus closed surfaces. Here, we assume that the adjacent circles used in circle packing are tangent to each other (see section “Discrete conformal deformation”); therefore, the edge length of [v_i_, v_j_] is determined by ^8.^

$${\text{l}}_{{{\text{ij}}}} = {\upgamma }_{{\text{i}}} + {\upgamma }_{{\text{j}}} .$$

### Surfaces of genus 2

We start from a triangular mesh of the closed surface in ${\mathbb{E}}^{3}$ to discuss our method since the triangularization of a smooth surface is the first step of discretization, and there are sophisticated tools available for this purpose^22^. In addition, since we only discuss discrete closed Riemann surfaces in our method, for conciseness, hereinafter, we refer to the discrete closed Riemann surfaces and discrete Gaussian curvature as the surface and Gaussian curvature, respectively, unless otherwise specified.

In section “Double torus”, we would use the surface of double torus^23^ to implement our theory and algorithm and provide a vivid illustration of the three major steps of our approach, which will be explained from sections “Pushing forward the triangular meshes to the parameter domain for genus 2” to “Generating textured surface for genus 2”, respectively.

In section “Vase vs. connecting rod”, the calculation of vase and connecting rod surfaces is carried out to testify the robustness and effectiveness of our method.

#### Double torus

##### Pushing forward the triangular meshes to the parameter domain for genus 2

**Fig. 1:**
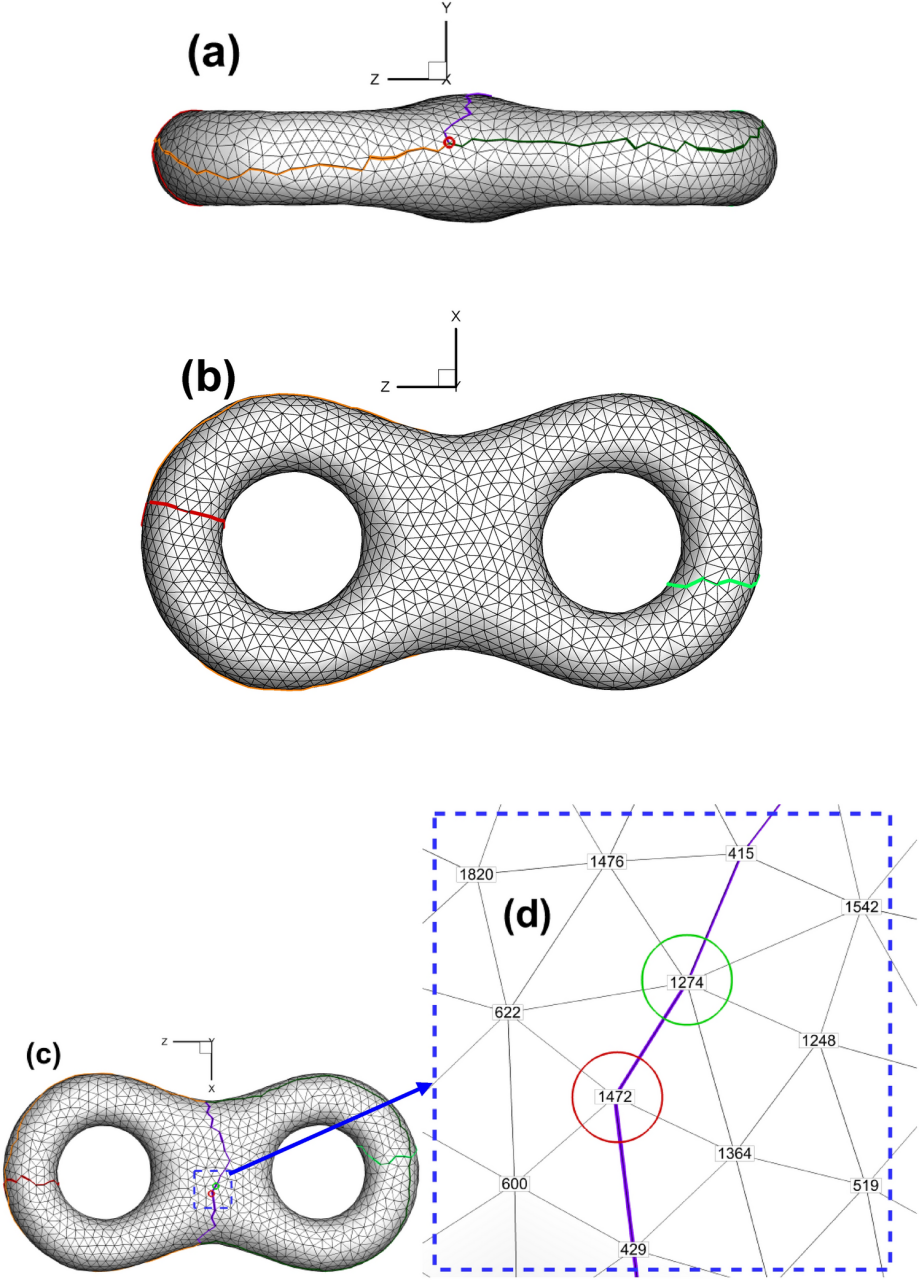
Triangular mesh of surface with cut graph. (**a**) Side view; (**b**) bottom view; (**c**) top view; (**d**) close-up view of mesh near the cut graph. (Also shown in Table 1, cut graph is composed of two handle loops (in red and light green) and three connection lines (in orange, dark green and purple) for genus 2.).

As shown in Fig. 1, we sketch a cut graph^8^ from the triangulated surface. The effect of the cut graph adopted in our method is the same as that in hyperbolic Ricci flow; namely, under these two circumstances, cutting the closed surface along the cut graph will lead to the fundamental domain^8^ of the original surface. However, as listed in Table 1, the compositions of the cut graphs in the above two application scenarios are slightly different. In hyperbolic Ricci flow, there are two kinds of loops, namely, handle loops and tunnel loops, whereas there is one kind of loop and one kind of connection line in our Euclidean Ricci flow situation (see Fig. 1 for details).

**Table 1:** Comparison of cut graph parameters in the Euclidean Ricci flow and the hyperbolic Ricci flow (surface of genus 2 and 3).

	The Euclidean Ricci flow (genus 2, genus 3)	The hyperbolic Ricci flow (genus 2, genus 3)
Number of tunnel loop	(0, 0)	(2, 3)
Number of handle loop	(2, 3)	(2, 3)
Number of connection line	(3, 6)	(0, 0)

Since the cut graph is well-known in algebraic topology^8^, we will not further elaborate here. We provide simplified names or abbreviations related to cut graphs in this paper to facilitate our future discussion. When cutting the surface along the cut graph, we will obtain new vertices induced by the vertex on the original surface. For convenience, we refer to the newly generated vertices as the child vertices of the original vertex. Conversely, the vertex on the cut graph of the original surface is called the father vertex of the newly generated vertices. Child vertices with the same father are called brother vertices to each other. As shown in Fig. 3, the orange, dark green and purple curves intersect, leading to an intersection vertex with three branches. Therefore, we call this the "3-way branching vertex". To particularly show the close relationship between the father vertex and its child vertices, we use the combination of child vertices to represent its father vertex on the original surface (for example, the combination of (C, G, N) and (K, P, J, L) denotes a 3-way branching vertex and a 4-way branching vertex in Fig. 3, respectively).

**Fig. 2:**
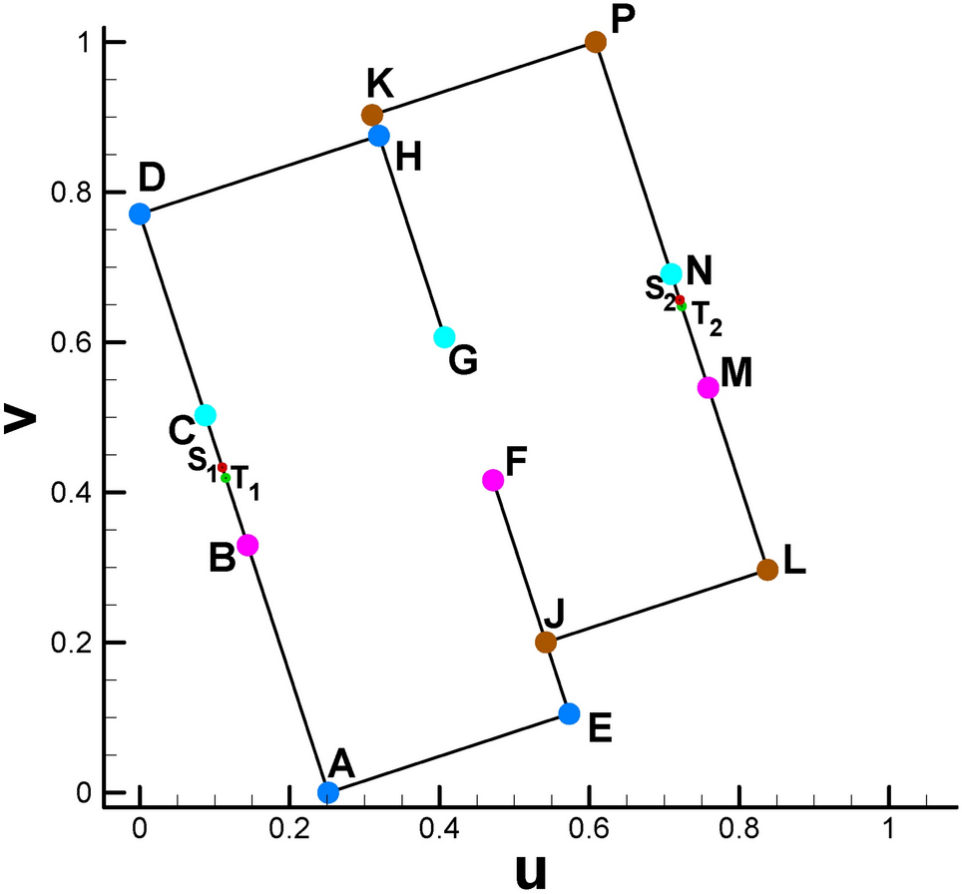
Illustration of spatial position of child vertices in the parameter domain (genus 2). Brother vertices are colored in the same color. Child vertices of 3-way branching vertex are C, G, N (cyan), B, F, M (purple). Child vertices of 4-way branching vertex are: A, E, H, D (light blue), K, P, J, L (brown).

Setting the target Gaussian curvature for child vertices in the parameter domain is the basis for subsequent steps, as shown in Fig. 2 and Table 2. Since each child vertex of the 4-way branching vertex will be at the corner of the rectangle, its Gaussian curvature is set to π/2. The three child vertices of the 3-way branching vertex, e.g., C, G and N, are set to 0, π and 0, respectively, which can be easily calculated through the angle deficit theory (see Eq. 2).

**Table 2:** Major control parameters and partial calculation results (genus 2).

Vertex	Vertex number	Gaussian curvature	(u, v)	Vertex	Vertex number	Gaussian curvature	(u, v)
A	1784	π/2	(0.2516, 0.0)	K	651	π/2	(0.3102, 0.9027)
B	1211	0	(0.1440,0.3297)	L	649	π/2	(0.8379, 0.2968)
C	919	0	(0.08752, 0.5027)	M	1210	0	(0.7587, 0.5394)
D	1785	π/2	(0, 0.7710)	N	920	0	(0.7093, 0.6908)
E	1787	π/2	(0.5731, 0.1049)	P	652	π/2	(0.6084, 1.0)
F	1212	-π	(0.4715, 0.4162)	S_1_	1596	0	(0.1102, 0.4332)
G	918	-π	(0.4068, 0.6067)	S_2_	1597	0	(0.7207, 0.6559)
H	1786	π/2	(0.3192, 0.8751)	T_1_	1379	0	(0.1147, 0.4196)
J	650	π/2	(0.5420, 0.2002)	T_2_	1378	0	(0.7233, 0.6479)

**Fig. 3:**
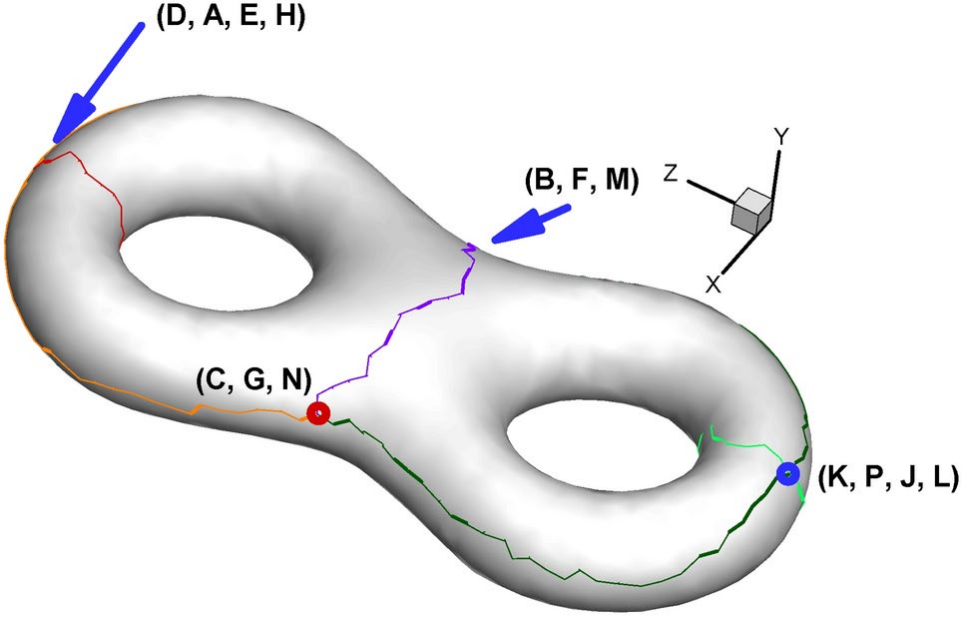
Illustration of 3-way branching vertex and 4-way branching vertex. There are two 4-way branching vertices and two 3-way branching vertices on the surface of genus 2. (Arrows are used to indicate the points on the far side of the surface and thereby invisible.).

Fig. 2 and Table 2 show the positions of the child vertices of the 3-way branching vertex and 4-way branching vertex in the parameter domain. Moreover, with the help of visualization of the parameter domain, we can gain deeper insight into the nature of 3-way branching vertices, helping us to better understand the singularity points of high genus surfaces (Fig. 3).

**Fig. 4:**
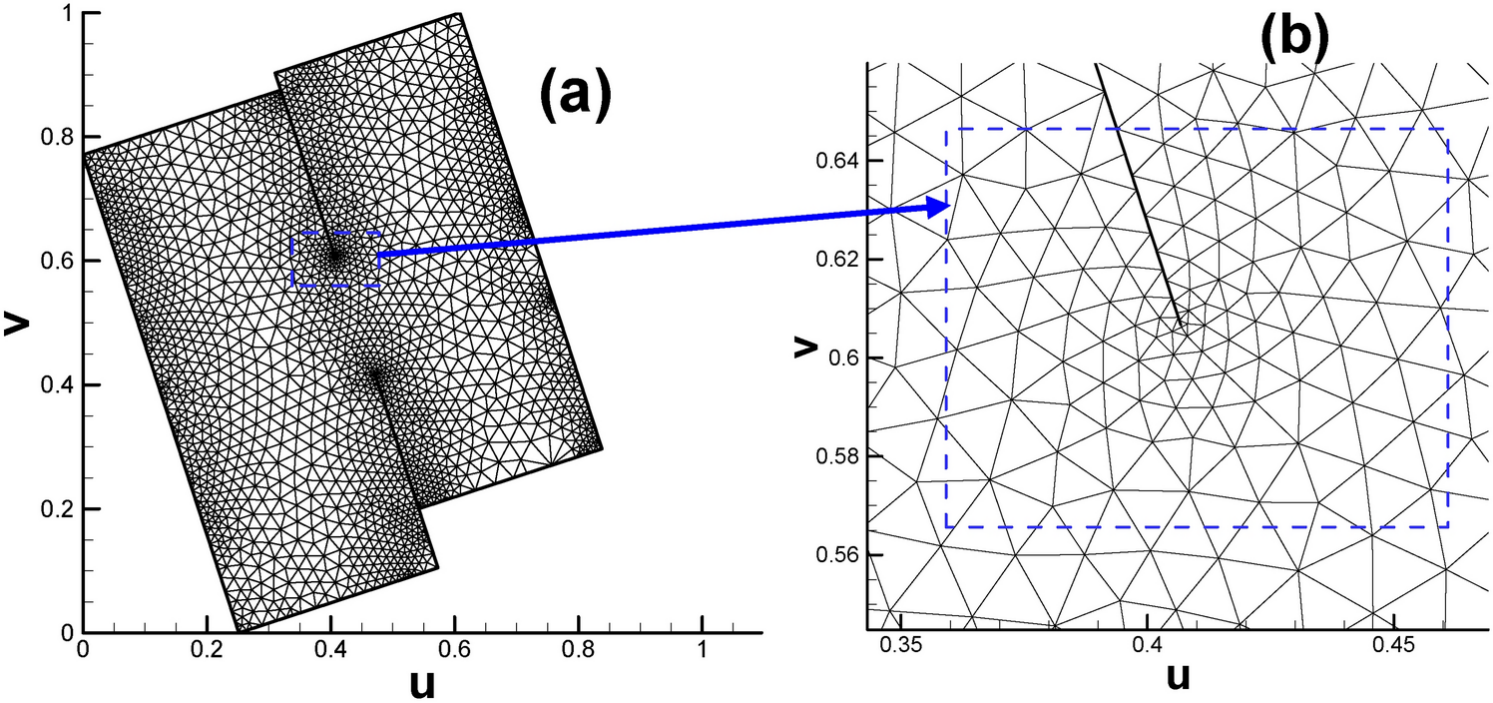
Embedding of triangular mesh of genus 2 surface in the parameter domain. (**a**) Overall view; (**b**) close-up view.

After assigning all the child vertices’ target curvatures listed in Table 2 (excluding S_1_, S_2_, T_1_ and T_2_ since they are not control points), we can proceed to calculate the Euclidean metric on the original surface and isometrically embed it in ${\mathbb{E}}^{2}$. Then, the prescribed fundamental domain of the high genus surface will take the form of two connected rectangles, as shown in Fig. 4a. And Fig. 4b enables us to gain deeper insight into the mesh near coincidence line.

In the past, only the Poincaré disk, Klein model or upper half-plane equipped with a special metric could embed the fundamental domain and hence display its real appearance, and we are very surprised to see that with the modification of the Euclidean Ricci flow, we managed to embed the fundamental domain of the genus 2 surface in ${\mathbb{E}}^{2}$. Subsequently, subsection “Pushing forward the triangular meshes to the parameter domain for genus 3” describes the process of embedding the fundamental domain of the genus 3 surface in ${\mathbb{E}}^{2}.$

After calculating the Euclidean metric on the original surface and subsequently embedding it in ${\mathbb{E}}^{2}$, the original curling and clustering child vertices on the original surface gradually separate from each other, as shown in Fig. 2.

For surfaces with genus >= 2, according to the Gauss-Bonnet theorem, there must be some singularity points for the parameterizations where the curvatures are not zero^9^. For the current situation, the 3-way branching vertex (C, G, N) in Fig. 3 is such a singularity point. By dividing the singularity point into several child vertices and assigning their corresponding Gaussian curvature (0, π, 0), we can embed them in ${\mathbb{E}}^{2}$. From a certain point of view, the reason why these points are called singularity points is that their neighborhood has to curl up on the original closed surface; by unfolding it in the parameter domain, we manage to turn the singularity points into regular points. In a sense, we uncover the mysterious cover of singularity points.

Moreover, the directions of rectangular edges AE and AD are not horizontal and vertical, respectively, because the whole embedded zone is determined by the choice of the first triangular cell (also called the root face ^9^) during the embedding process. More precisely, the fact that we usually make the first edge of the root face parallel to the x-axis of ${\mathbb{E}}^{2}$ when embedding makes the embedding result of the whole parameter domain vary dramatically with different choices for selecting the root face. However, once the embedded rectangular zone is determined, for convenience, we will still call the lines AE and AD the horizontal and vertical directions, respectively. Here, we clarify that in most situations of this paper, the two expressions of “parameter domain” and “fundamental domain” are considered identical, and both of them can be used to call the embedded rectangular zone. However, since our focus will be on designing the parameters on the prescribed rectangular zone, for convenience, we call this the parameter domain instead of the fundamental domain unless otherwise stated.

##### Design of the checkerboard texture in the parameter domain

Following the general practice of visualizing the vector field of the original closed surface, we adopt the checkerboard texture mapping method for display. On the micro scale, the checkerboard texture can be viewed as a combination of quadrilateral cells.

**Fig. 5:**
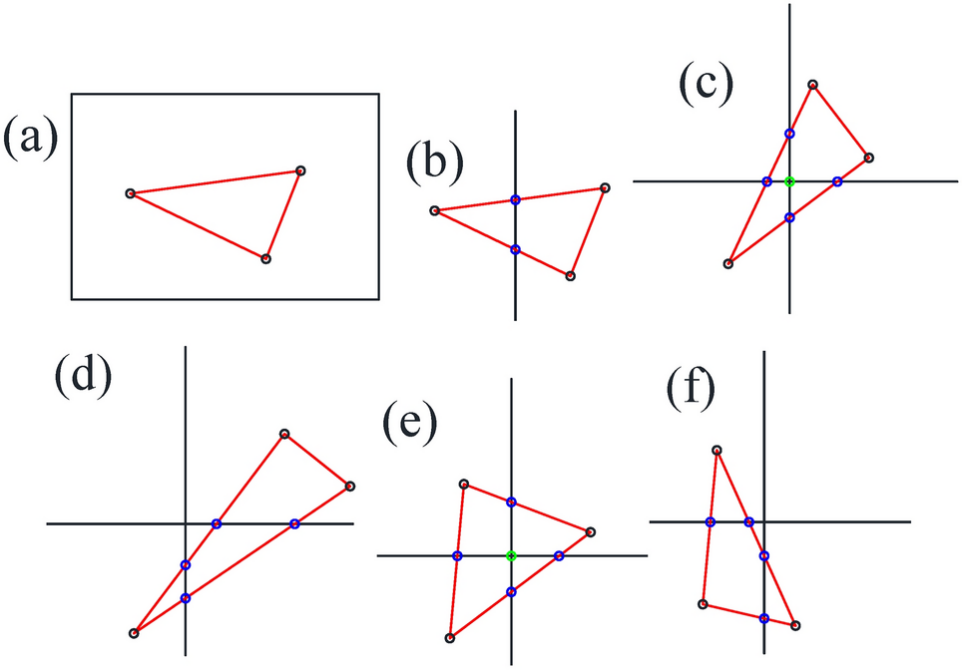
Typical spatial position relation between quadrilateral cells and triangular cells when the former are dramatically larger than the latter. In case (**a**), a triangular cell is completely within the scope of a quadrilateral cell. In case (**b**) to (**f**), there are intersection points between quadrilateral cells and a triangular cell. The quadrilateral cell vertices (light green circle) and intersection points (blue circle) will be calculated by their barycentric coordinates within the corresponding triangular cell.

Fig. 5 shows the typical position relation between the quadrilateral cell and triangular cell when the former is dramatically larger than the latter. In more complicated cases when the two types of cells have similar sizes, their spatial position relation can be deduced and extended from the basic case shown in Fig. 5. In summary, by calculating the intersection points of the edges of quadrilateral cells and triangular cells, we can obtain subdivided triangles or polygons based on the original triangular cell.

**Fig. 6:**
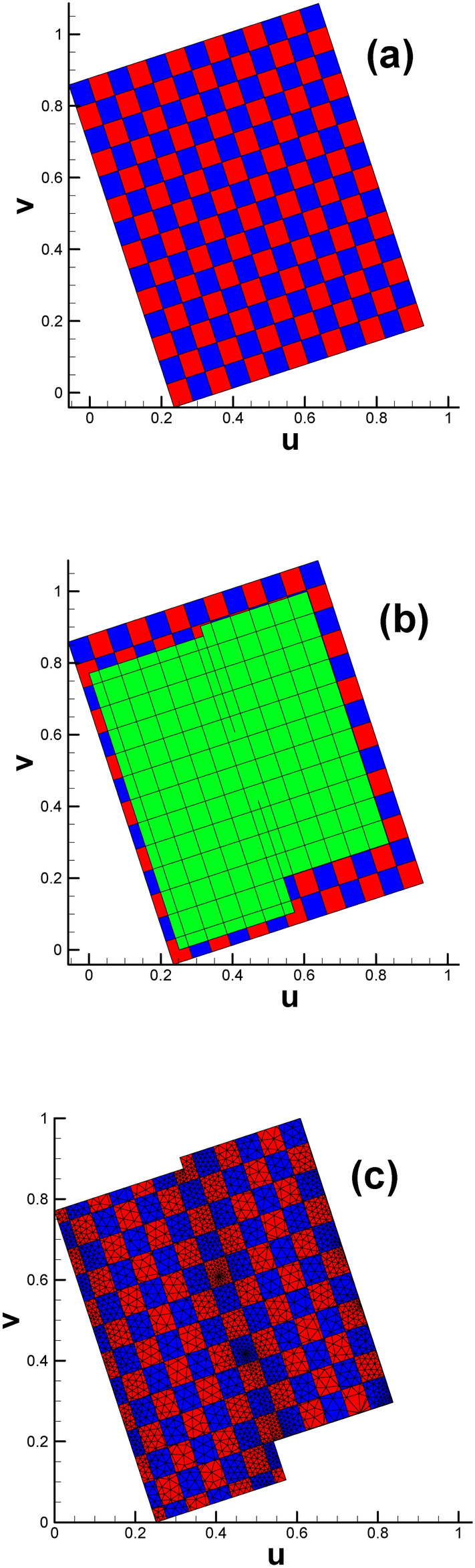
Subdividing triangular mesh with quadrilateral cells of classical checkerboard texture. (**a**) Classical checkerboard texture; (**b**) triangular mesh with classical checkerboard texture; (**c**) triangular mesh in the parameter domain after being subdivided.

We initially intend to use classical checkerboard textures with evenly distributed contour lines for both horizontal and vertical directions to parameterize the domain; see Fig. 6a. Using the subdivision technique described in Fig. 5, we obtain the distribution of multiple triangles and polygons, as shown in Fig. 6c. However, this type of checkerboard setting will lead to mismatching of the texture at the border when pulling back to the original surface (see Fig. 11 in subsection “Generating textured surface for genus 2”).

The reason is that the straight line linking two brother vertices (e.g., S_1_ and S_2_ in Fig. 2) is not always parallel to the AE line for horizontal isoparametric curves. This fact is also valid for vertical circumstances. Therefore, the setting of an evenly distributed texture is not suitable for the internal property of the parameter domain. Similar to the setting of periodic boundary conditions in fluid dynamics, we attempt to develop a method to specifically handle the mismatch problem at the boundary of the parameter domain accordingly, which can be summarized as follows. It is very easy to verify or infer from continuity theory that when we randomly select two adjacent vertices on the cut graph of the original surface, their child vertices must be adjacent to each other. Let us take an example from the mapping result from the original surface to its parameter domain. As shown in Fig. 1d, we randomly choose two vertices No. 1472 (vertex S) and No. 1274 (vertex T) on the cut graph. After calculating, we can find their child vertex pairs (S_1_, T_1_) and (S_2_, T_2_) next to each other on the boundaries of the parameter domain shown in Fig. 2.

At this stage, although we have almost finished the process, there is still a concern to be addressed. That is, whether these isoparametric curves of the same family obtained by the above method will intersect with one another. We can determine the answer to this question because conformal transformation preserves the property of diffeomorphism.

**Fig. 7:**
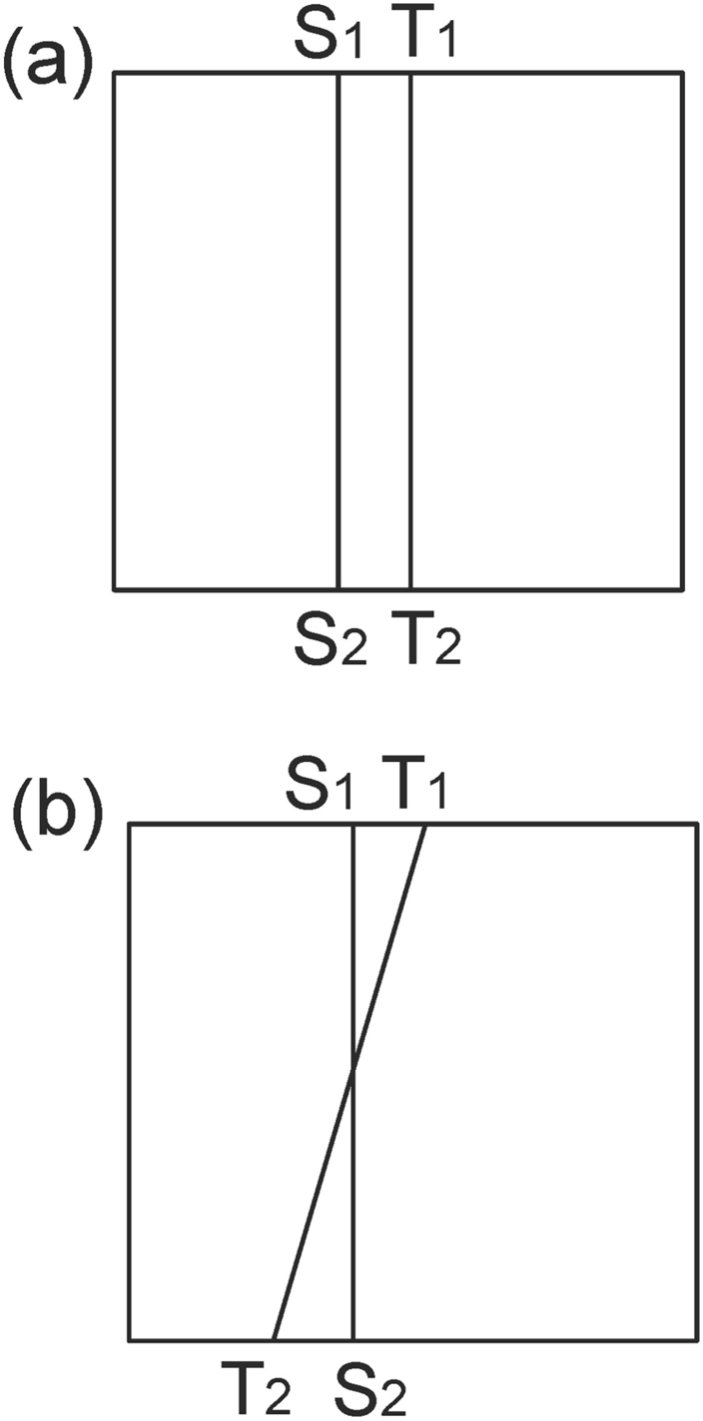
Spatial position relationship between the newly obtained child vertices. (**a**) Preserving the upstream and downstream relationship between their father vertices; (**b**) not preserving the upstream and downstream relationship between their father vertices.

We can provide a brief overview of this. The transformation via surface Ricci flow is a conformal mapping process^18^, making it biholomorphically diffeomorphic between the original surface and its parameter domain^9^. We draw two straight lines to link the S_1_ and S_2_ vertices and the T_1_ and T_2_ vertices. We assign a tangent vector for each point along the two lines, supposing that the situation in Fig. 7b holds, then there will be an intersection point induced by the intersection of two lines where there is no definite direction, contradicting the requirement of maintaining the diffeomorphism property of conformal mapping.

Therefore, child vertices preserve their fathers’ upstream and downstream relative position relations. The fact that Fig. 7a holds paves the way for manually designing the texture in the parameter domain: when we design the spatial position of the pair of brother vertices on the boundary of the parameter domain, then the internal texture can be determined by drawing lines to link the two brother vertices.

After addressing this major concern, the following three minor issues must be addressed: (a) degenerated triangular cells, (b) coincidence lines and (c) 3-way branching vertices.

**Fig. 8:**
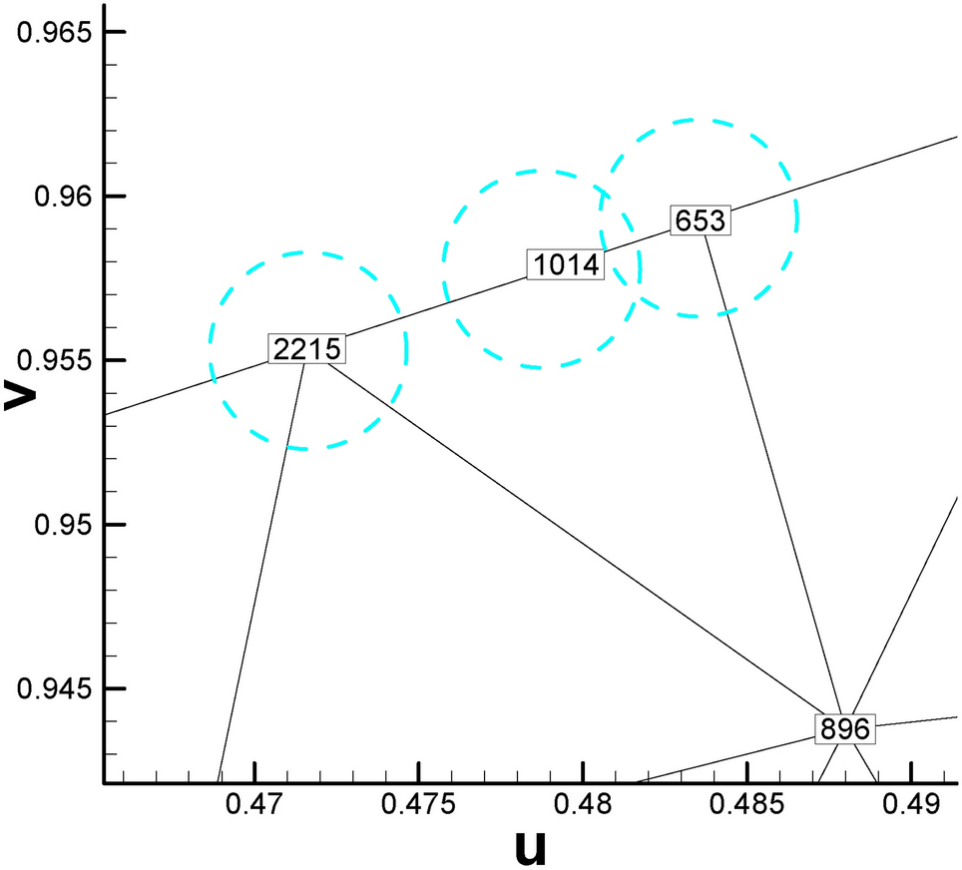
Illustration of three vertices of a triangular cell being collinear. (The three vertices No. 2215, No. 1014 and No. 653 are roughly in the middle of KP line, as shown in Fig. 2.).

**Fig. 9:**
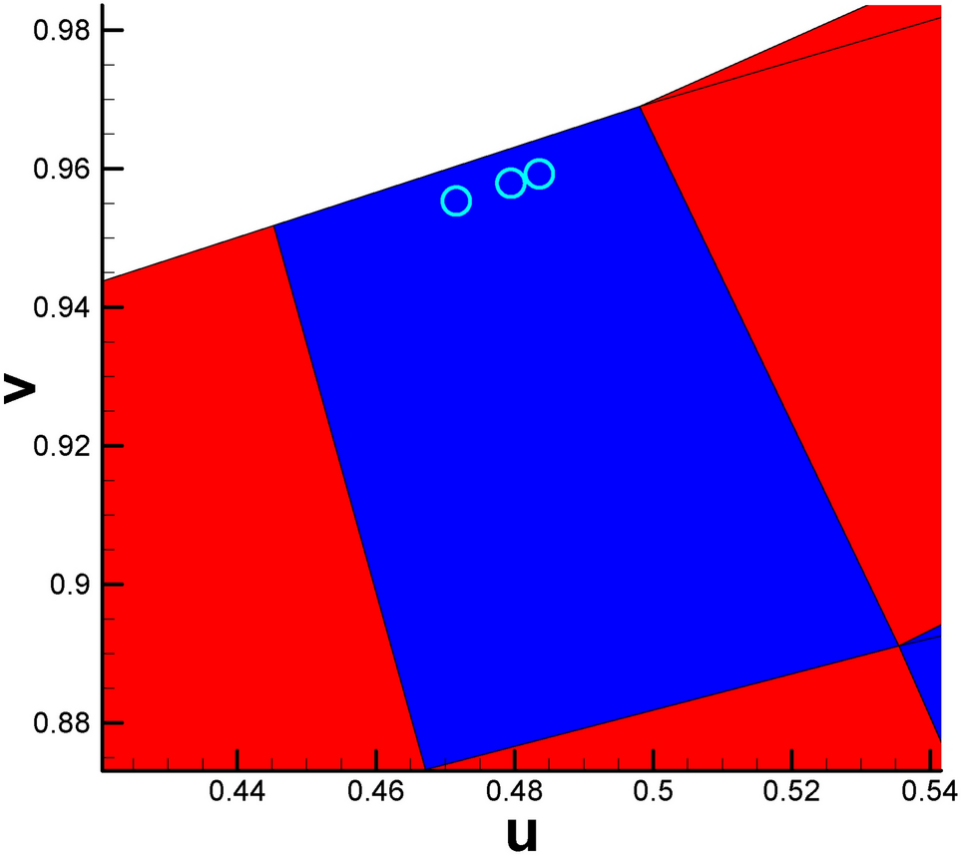
Illustration of three vertices on a straight line completely covered by a quadrilateral cell.

**Fig. 10:**
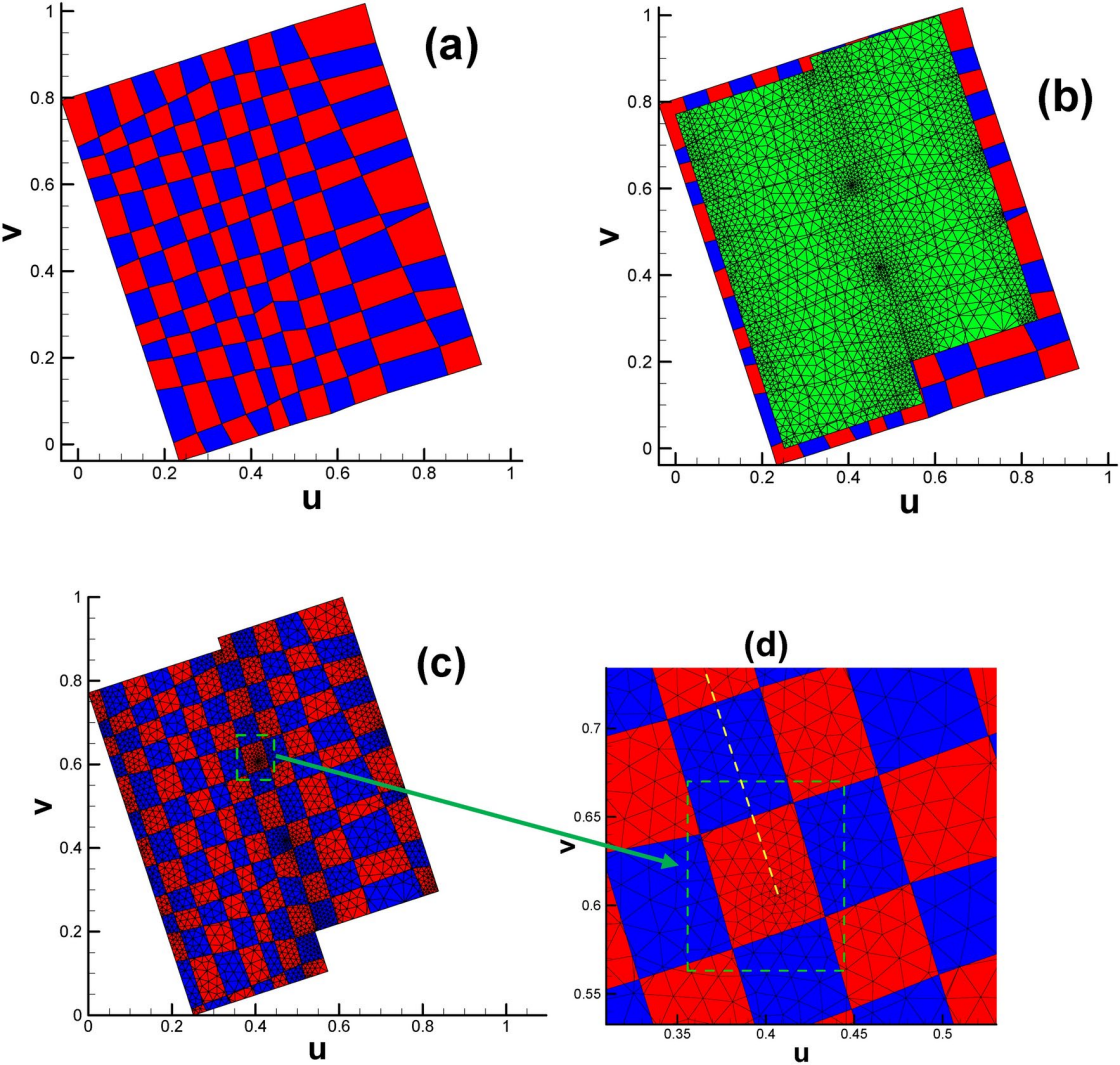
Illustration of subdividing triangular mesh with quadrilateral cells of modified checkerboard texture (genus 2). (**a**) Modified checkerboard texture; (**b**) triangular mesh with modified checkerboard texture; (**c**) triangular mesh in the parameter domain after being subdivided; (**d**) close-up view of the mesh near the coincidence line, highlighted in vivid yellow.

- Occasionally, there might be some degenerated triangular cells during the Euclidean Ricci flow mapping process. Specifically, three vertices of the triangular cell in the parameter domain are on a straight line (see Fig. 8). In this situation, we recommend allowing one quadrilateral cell to completely cover the three vertices belonging to the same triangular cell (see Fig. 9) to avoid the possible discontinuity of texture on the original surface.

(b) Fig. 2 shows that there is a partial coincidence between straight lines GH and GK. However, when magnifying the coincidence line many times, we can see that the two lines are still separated by a tiny gap inevitably introduced by computer numerical error. To ensure the continuity of the texture, we use one quadrilateral cell to stretch across or bridge the gap. Therefore, the two sides of the coincidence line are set to the same color (see Fig. 10d).

(c) In practice, handling 3-way branching vertices is much more difficult than handling regular vertices when designing checkerboard textures. The reason is that the neighborhoods of the child vertices of the 3-way branching vertex are disjoint in the parameter domain (see the property of deck transformation^24^), while their colors must be in accord with one another at the same time. Therefore, in designing the texture, we need to make every quadrilateral cell covering one child vertex compatible with another two quadrilateral cells in terms of color and spatial positioning.

Having taken into consideration all three issues listed above, we managed to obtain a designed checkerboard texture, which is slightly larger than the parameter domain of the original surface (see Fig. 10a and b). By intersecting the larger texture zone with the parameter domain, we obtain the textured triangular mesh (see Fig. 10c).

We simply use piecewise linear curves to guarantee the periodic boundary condition without paying much attention to the beauty of the texture. Interested readers can further explore how to overcome this limitation.

##### Generating textured surface for genus 2

**Fig. 11:**
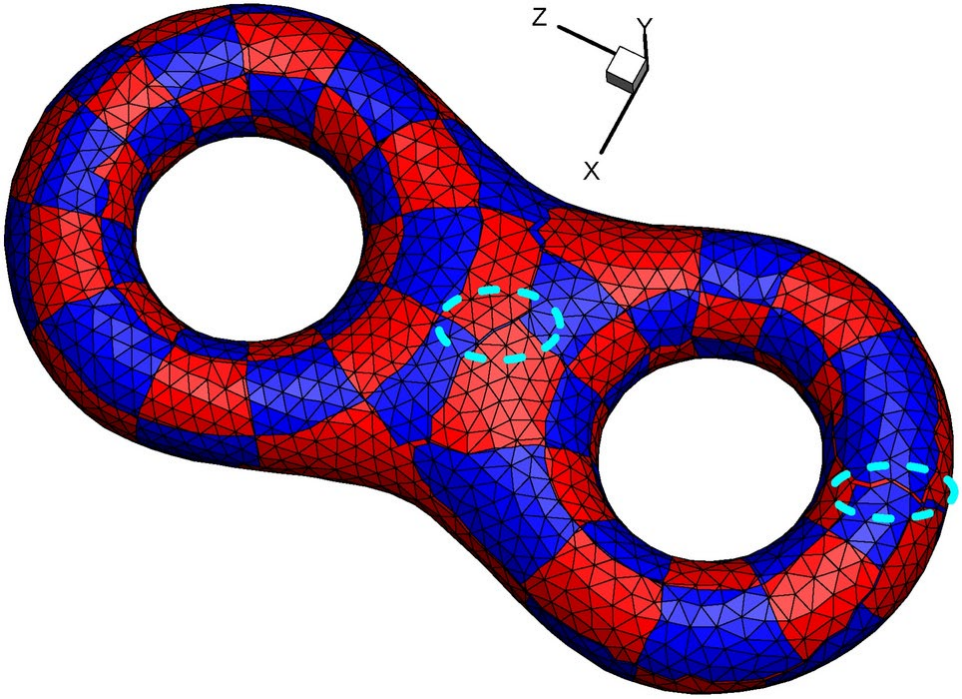
Illustration of the mismatch of texture on the original surface.

By pulling back the classical checkerboard texture shown in Fig. 6, we obtain the textured surface shown in Fig. 11. However, there are several places where the texture could not match smoothly and continuously, as sketched with the cyan dashed line. Therefore, we follow the modified design of texture shown in Fig. 10.

Just as holomorphic 1-form induces an affine atlas covering the whole surface except for several zero points^8^, after unfolding the neighborhood of the singularity point, we can take advantage of the affine structure to pull the checkerboard texture back to its original surface. More precisely, since affine transformations preserve the barycentric coordinates^25^, we can use barycentric coordinates to linearly interpolate the quadrilateral cell vertices and the intersection points (shown in Fig. 5) by the three vertices of the triangular cell involved.

**Fig. 12:**
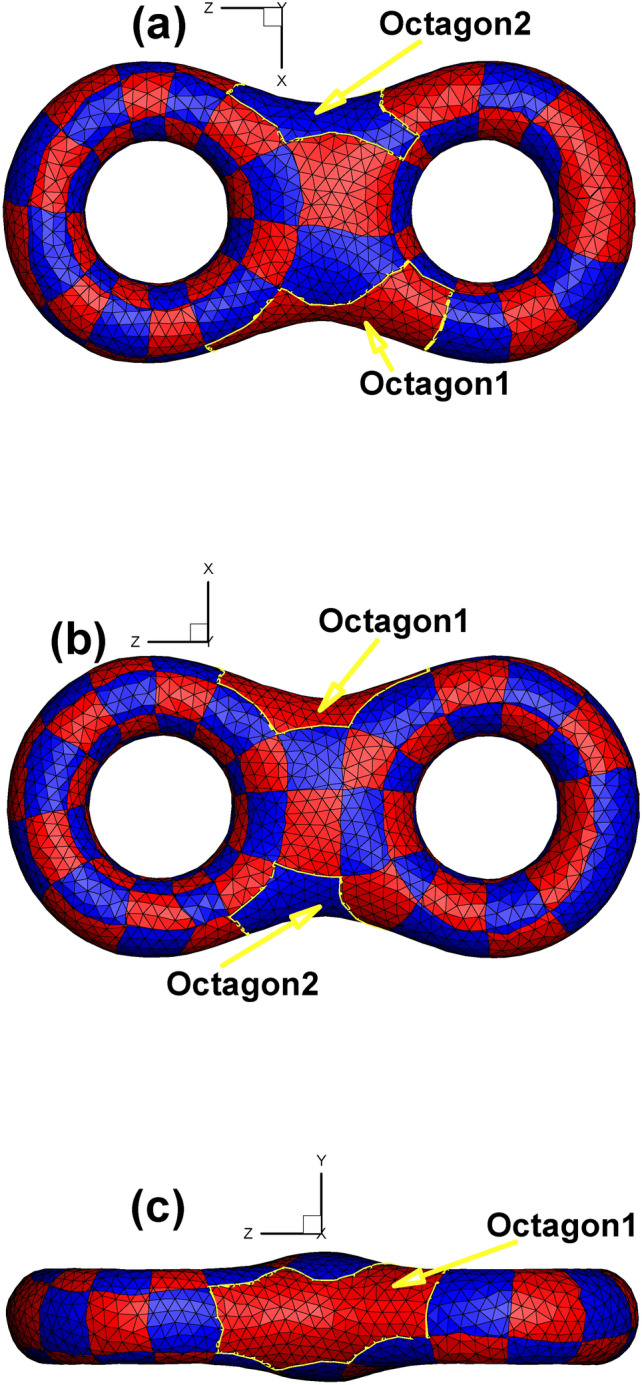
Checkerboard textured surface of genus 2 by Euclidean Ricci flow. (**a**) Bottom view; (**b**) top view; (**c**) side view.

Based on the technical route of Euclidean Ricci flow rather than the holomorphic 1-form method^26^, we also obtain two octagons on the textured surface. To highlight the octagons, we use the vivid golden color to show their outlines (see Fig. 12). In addition, the images obtained by our method are vector diagrams and preserve the image clarity after being magnified many times, which is much better than the general practice of using bitmaps in the parameter domain^15^ to show the texture.

Moreover, the images obtained by our method is vector diagram and preserves the image clarity after magnifying many times (see Fig. 12), which is different from the general practice to use bitmap in the parameter domain to show the texture^15^.

**Fig. 13:**
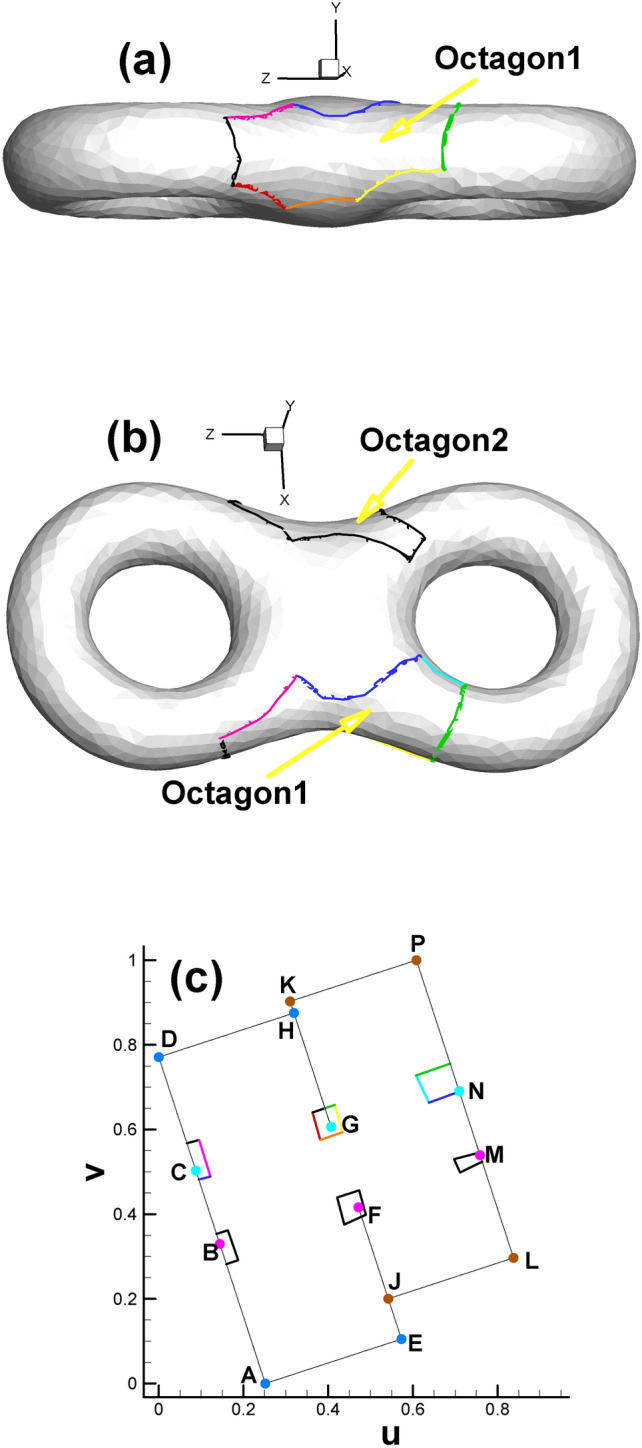
Illustration of the outline of one octagon on the original surface using rainbow colors. (**a**) and (**b**) are two views of the same surface that attempt to completely show the entire 8 sides of the octagon. (**c**) is an image of the octagon in the parameter domain.

Table 3 presents detailed information of every child vertex and its father. As shown in Fig. 13, to have a noticeable effect, we omit the meshes and use the 7 colors of the rainbow and one black color to show the total of 8 edges of one octagon (namely, the “Octagon1” in Fig. 12 to serve as a representative). Specifically, the color sequence of the rainbow from red to orange to purple to black is used to indicate the first to the eighth edge of the octagon. Furthermore, two consecutive colors in the rainbow sequence indicate two adjacent edges. Therefore, for example, the red, orange and yellow edges are the first three edges of the octagon. The above rules for setting colors hold for both the original surface and its corresponding parameter domain.

**Table 3:** Child vertex information shown in Fig. 13.

Name of child vertex	Color of child vertex	Its Father vertex	Location of its father vertex
C	Cyan	(C, G, N) in Fig. 3	Octagon1 in Fig. 13b
G	Cyan	(C, G, N) in Fig. 3	Octagon1 in Fig. 13b
N	Cyan	(C, G, N) in Fig. 3	Octagon1 in Fig. 13b
B	Pink	(B, F, M) in Fig. 3	Octagon2 in Fig. 13b
F	Pink	(B, F, M) in Fig. 3	Octagon2 in Fig. 13b
M	Pink	(B, F, M) in Fig. 3	Octagon2 in Fig. 13b

Since the octagon covers the 3-way branching vertex shown in Fig. 3, we can take advantage of Figs. 3 and  13 to briefly state the concept of double cover within the scope of complex analysis^27^. Assuming that we fix a vector on the 3-way branching vertex (C, G, N), when this vector rotates 360° on the original surface around its initial point, the vector’s image will rotate 720° in the parameter domain (see Fig. 13c, which shows 360° from GH to GK, 180° from NP to NI, and again 180° from CD to CA). In doing so, the neighborhood of child vertices in the parameter domain can be viewed as a double cover of that of a 3-way branching vertex, providing another perspective from which to view the process of turning singularity points to regular points.

#### Vase vs. connecting rod

**Fig. 14:**
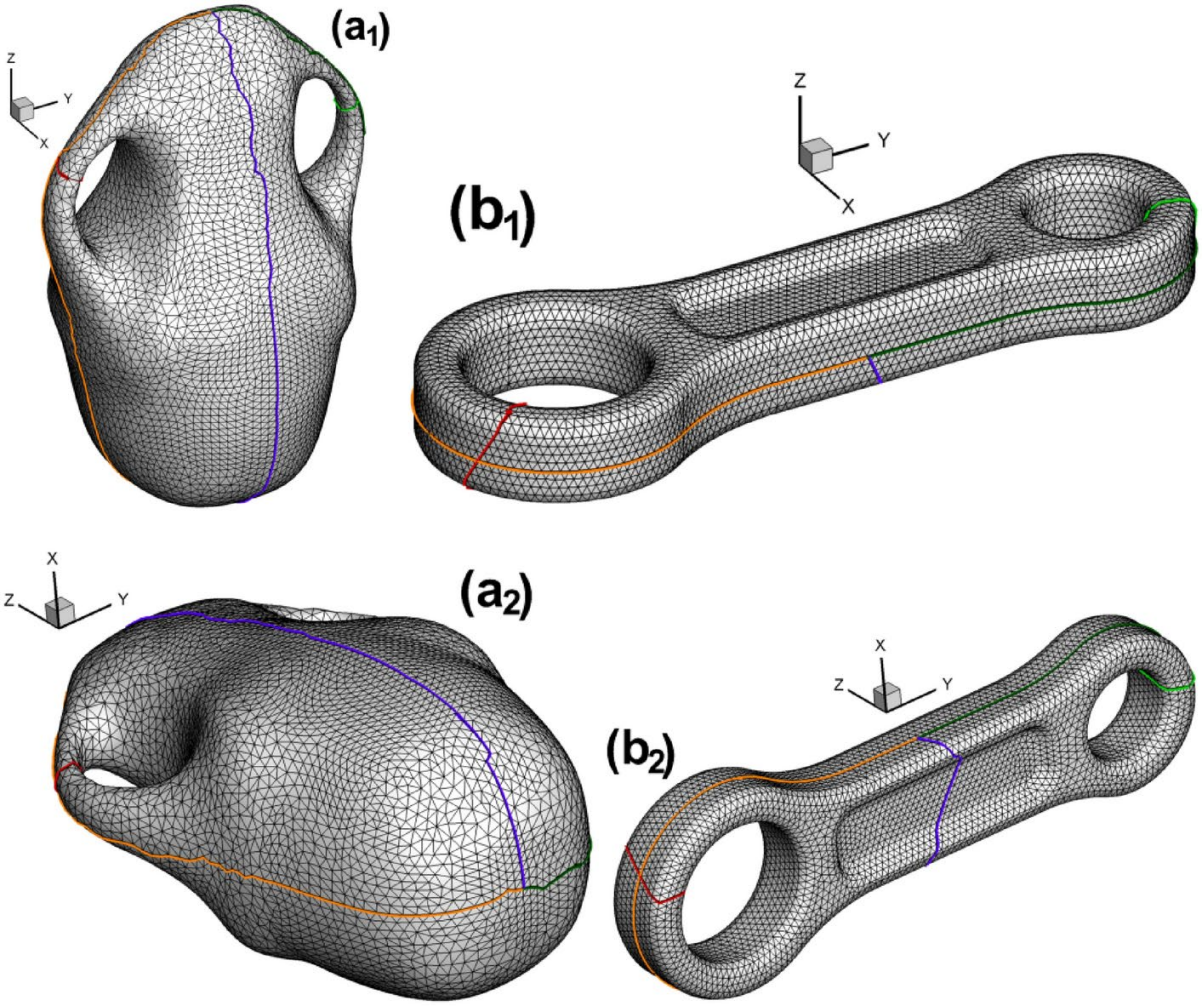
Triangular mesh of vase and connecting rod with cut graph. (**a**_**1**_) Vase in isometric view1; (**b**_**1**_) connecting rod in isometric view1; (**a**_**2**_) vase in isometric view2; (**b**_**2**_) connecting rod in isometric view2.

As shown in Fig. 14, we sketch cut graphs for triangulated vase surface and connecting rod surface, respectively.

**Fig. 15:**
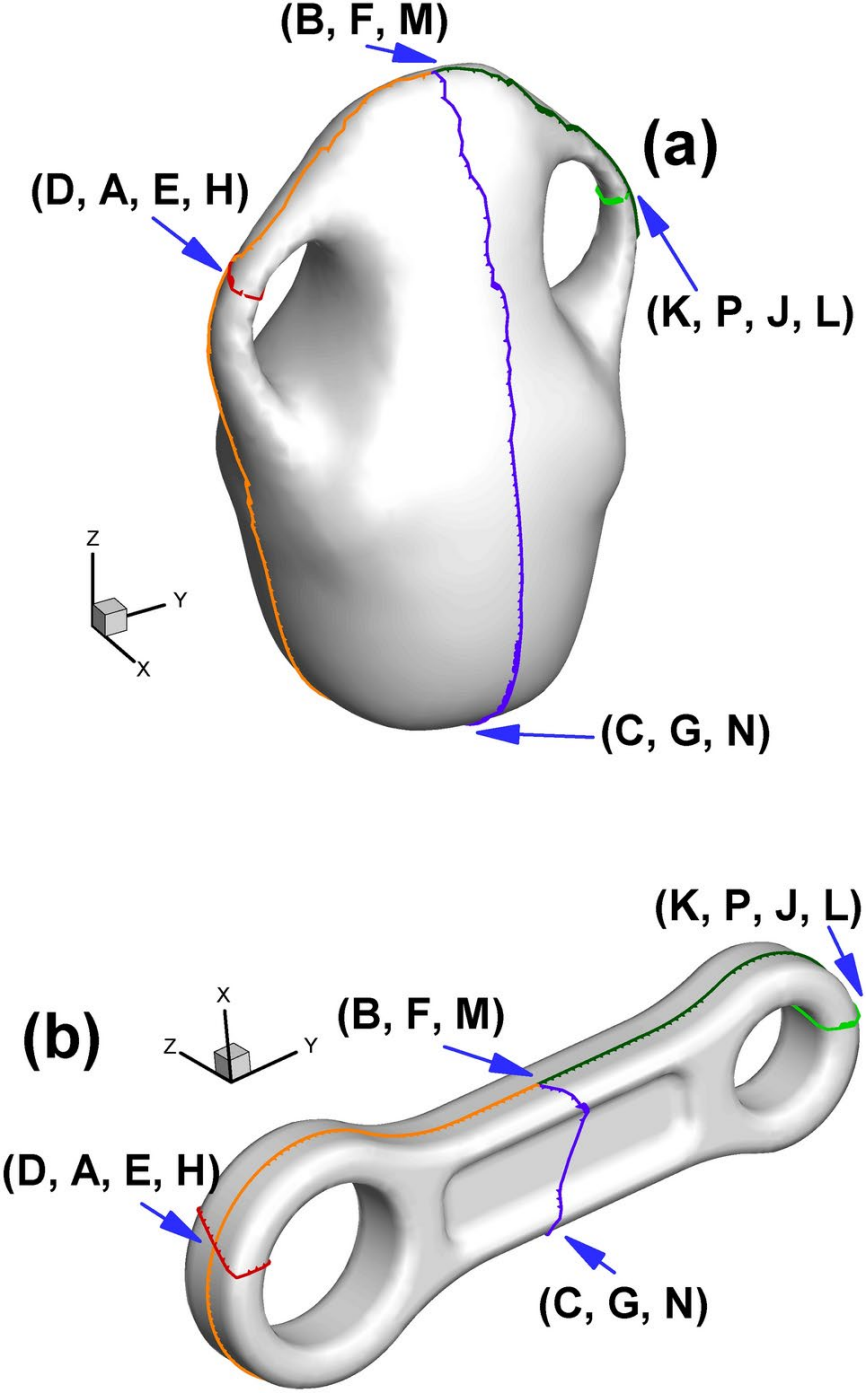
Illustration of 3-way branching vertex and 4-way branching vertex and their child vertices. (**a**) Vase; (**b**) connecting rod.

After cutting the closed surface along the cut graph, the child vertices of the original vertex could be obtained, shown in Fig. 15.

**Fig. 16:**
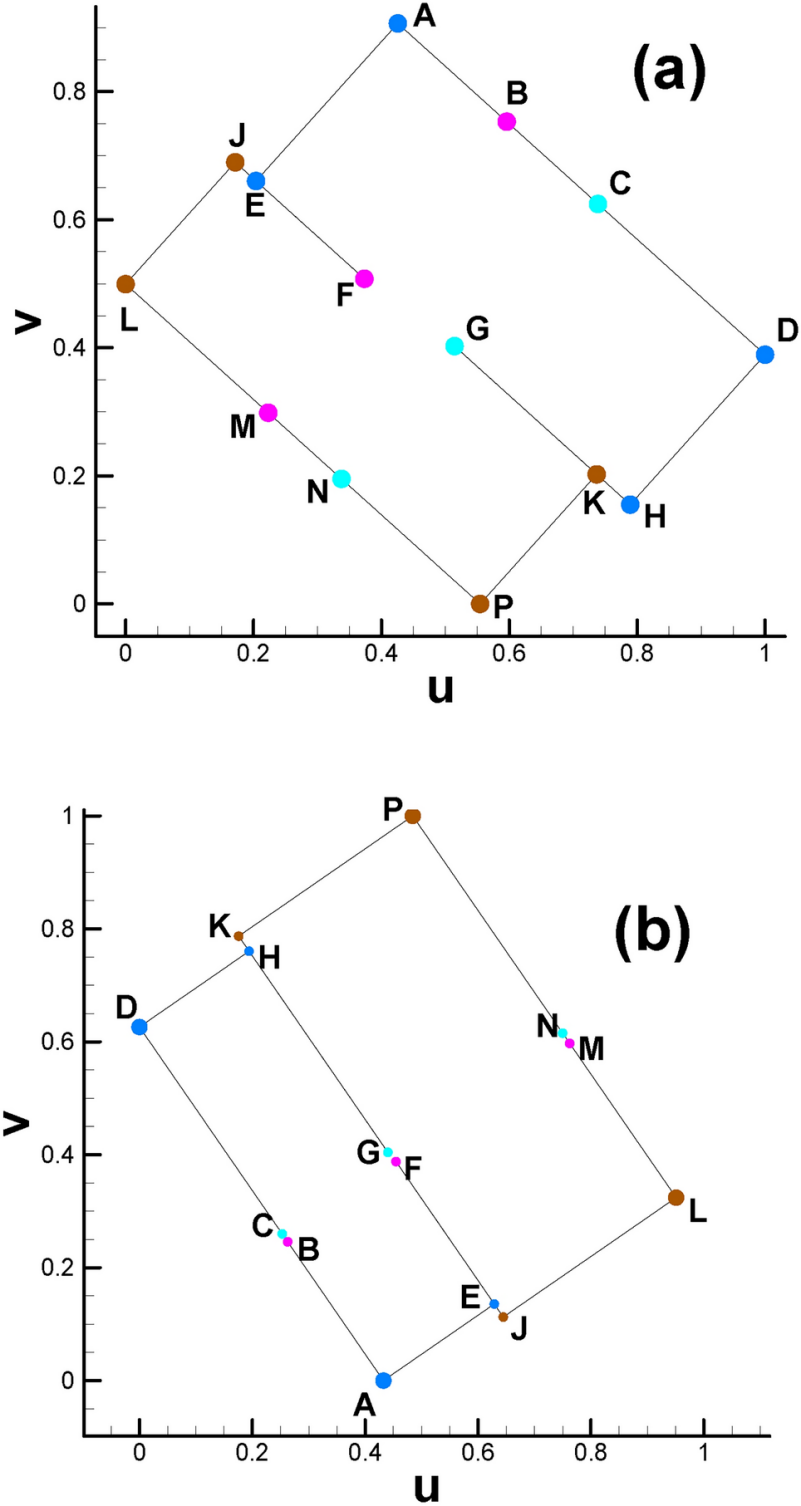
Illustration of spatial position of child vertices in the parameter domain. (**a**) Vase; (**b**) connecting rod.

 Fig.16 shows the positions of the child vertices of the 3-way branching vertex and 4-way branching vertex in the parameter domain for vase and connecting rod, respectively. It can be seen that the horizontal and vertical directions for the two cases are not the same. The reason is that the choice of the first triangular cell to embed for the two cases is different, which has been stated in subsection  “Pushing forward the triangular meshes to the parameter domain for genus 2”.

**Fig. 17:**
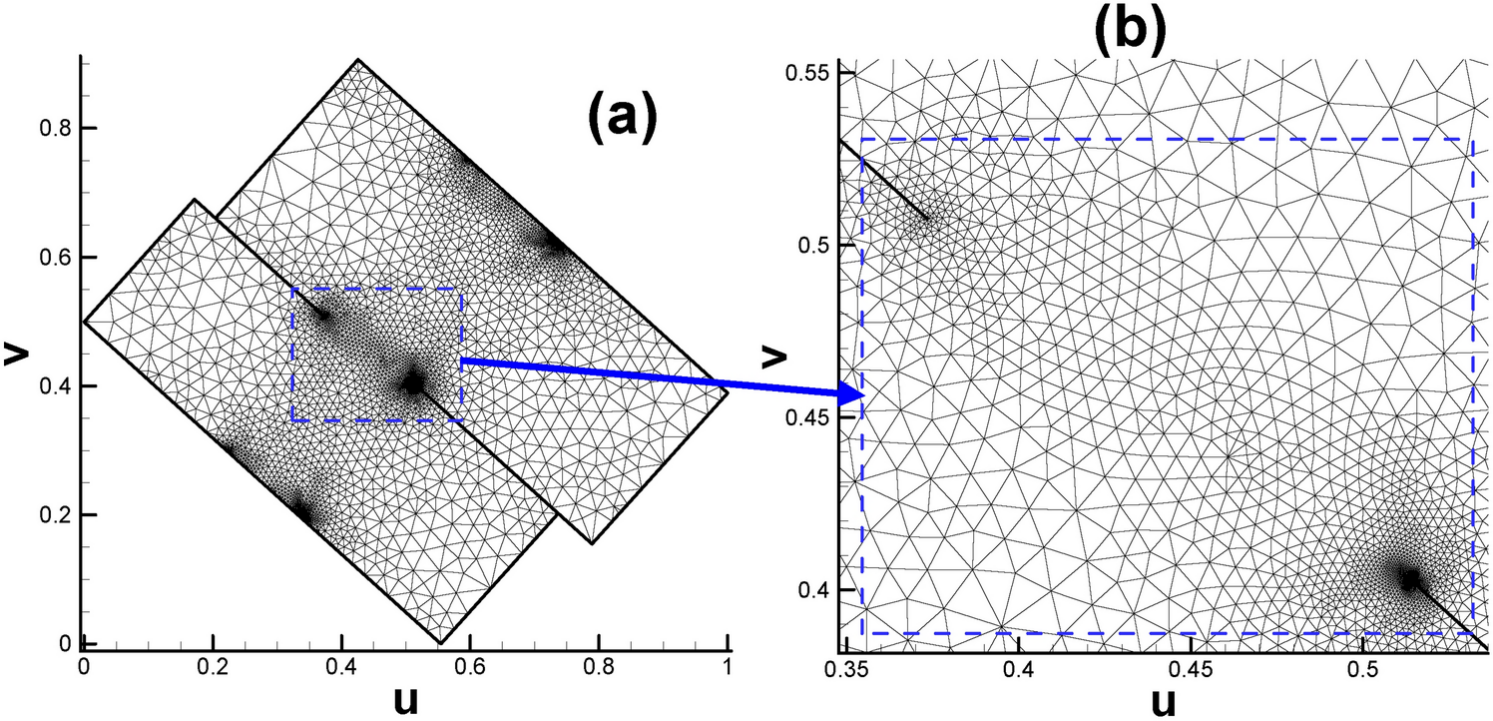
Embedding of triangular mesh of vase surface in the parameter domain. (**a**) Overall view; (**b**) close-up view.

**Fig. 18:**
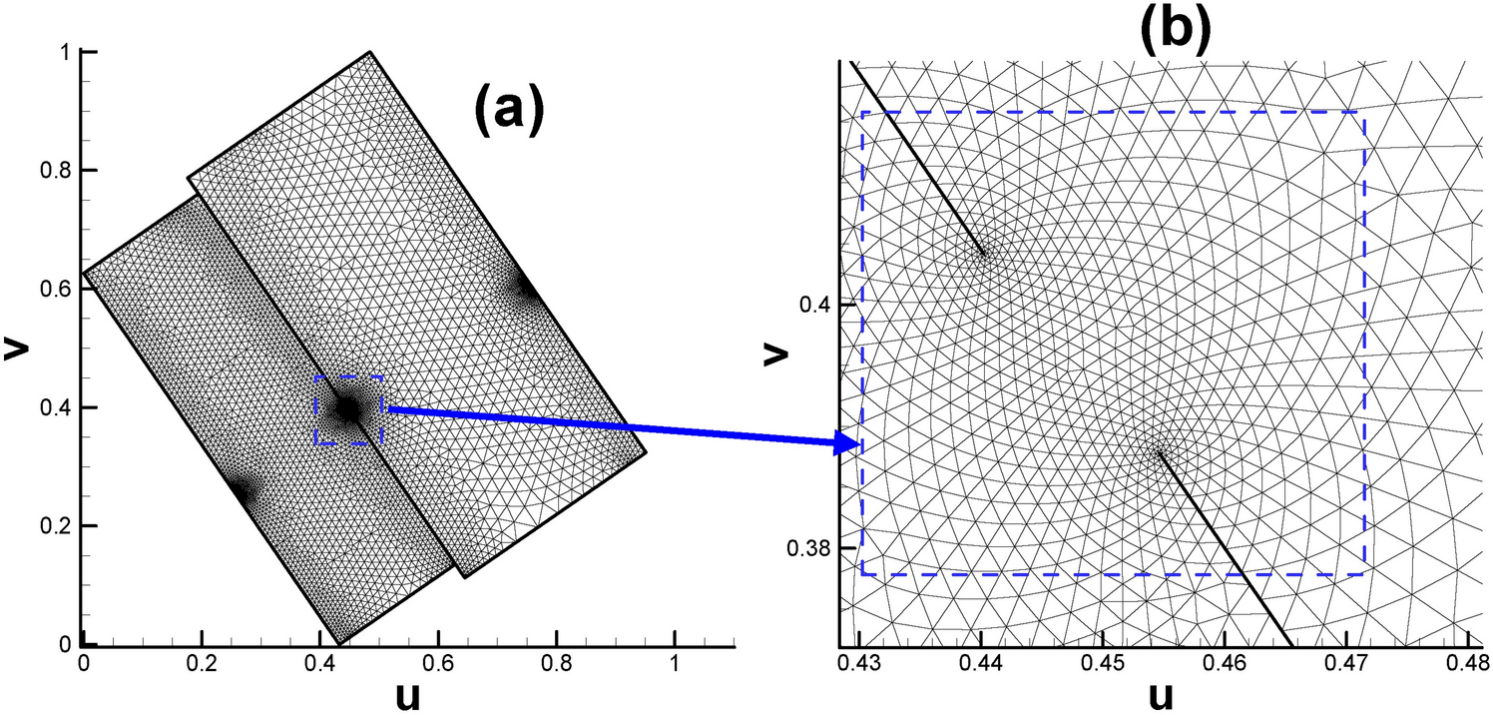
Embedding of triangular mesh of connecting rod surface in the parameter domain. (**a**) Overall view; (**b**) close-up view.

The fundamental domain of the vase and connecting rod surface will both take the form of two connected rectangles, as shown in Figs. 17a and 18a, respectively. While Figs. 17b and 18b enable us to gain deeper insight into the mesh near coincidence line for the two calculation cases.

**Fig. 19:**
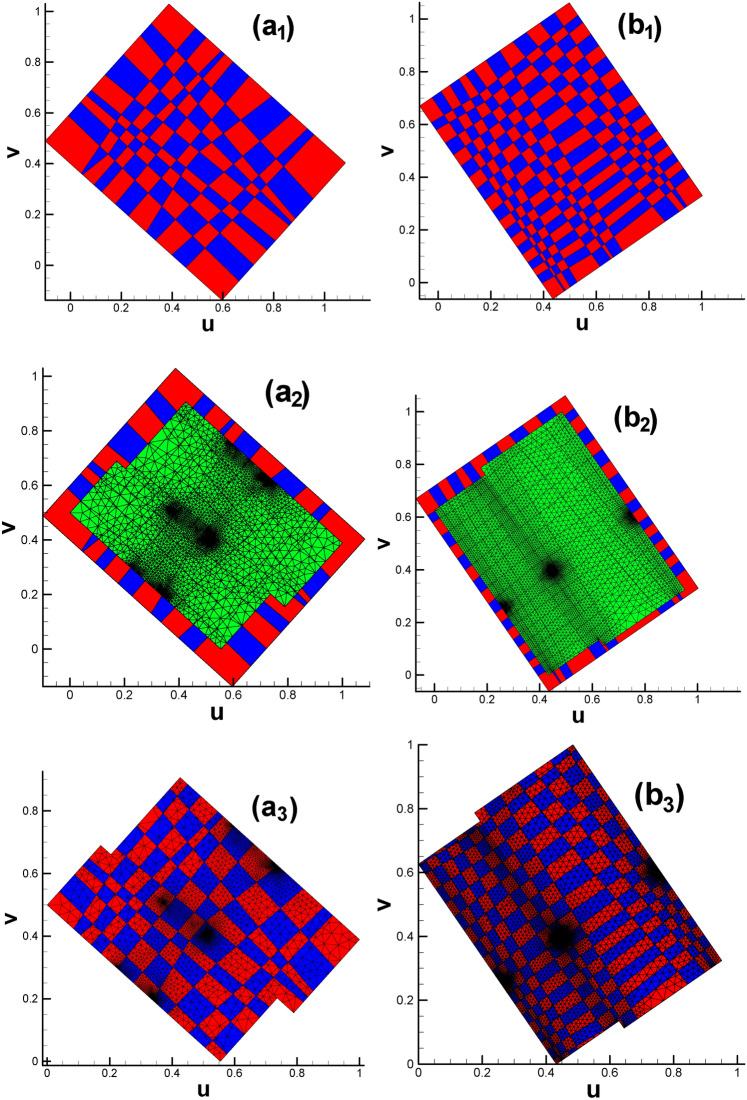
Subdividing triangular mesh with quadrilateral cells of classical checkerboard texture. (**a**_**1**_)–(**a**_**3**_) Classical checkerboard texture, triangular mesh with checkerboard texture and triangular mesh after being subdivided for vase; (**b**_**1**_)–(**b**_**3**_) classical checkerboard texture, triangular mesh with checkerboard texture and triangular mesh after being subdivided for connecting rod.

Subsequently, two checkerboard textures are designed for vase and connecting rod (see Fig. 19(a_1_) and (b_1_)), respectively. By intersecting the larger texture zone with the parameter domain, we obtain the textured triangular mesh (see Fig. 19(a_3_) and (b_3_)). To ensure the continuity of texture on the original surface, it is recommended to make the child vertices of 4-way branching vertex and 3-way branching vertex located in the same color cell in the checkerboard, see Fig. 19(a_2_) and (b_2_).

**Fig. 20:**
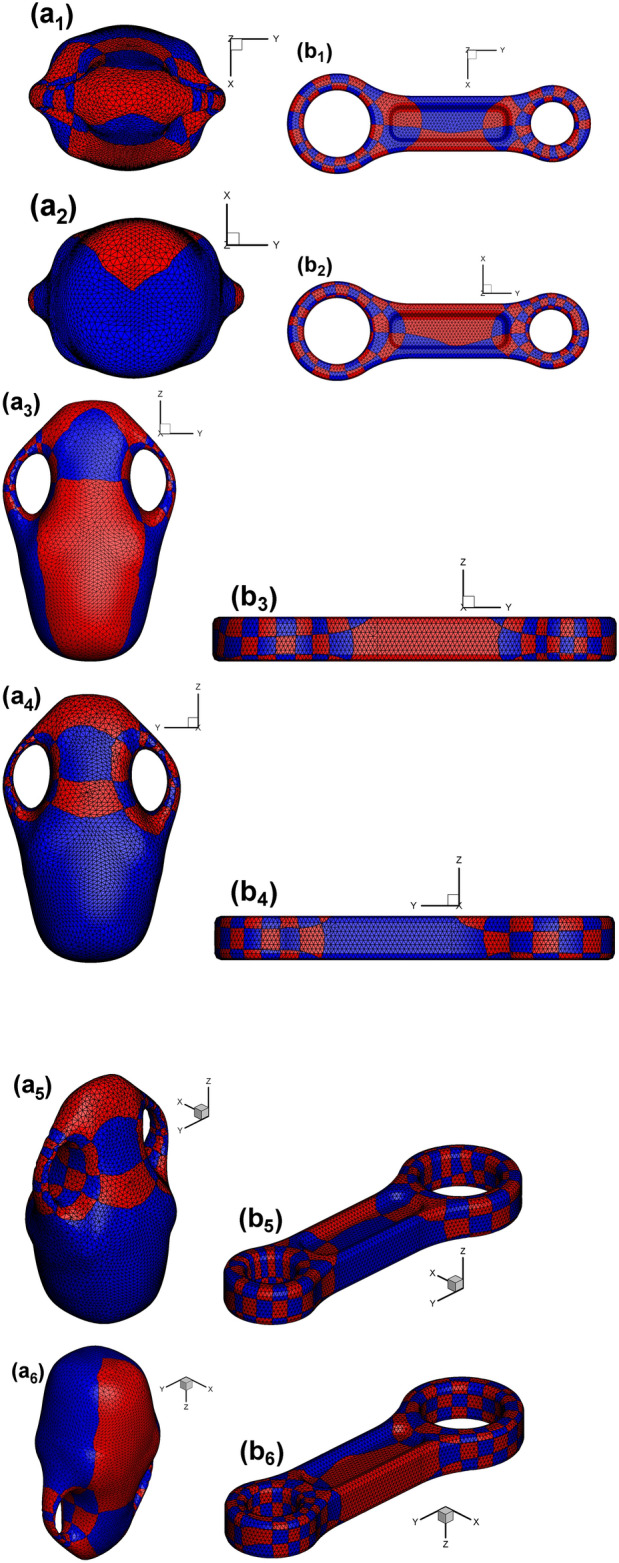
Checkerboard textured surface by Euclidean Ricci flow. (**a**_**1**_)–(**a**_**4**_) Vase in four oriented views, (**a**_**5**_),  (**a**_**6**_) vase in two isometric views; (**b**_**1**_)–(**b**_**4**_) connecting rod in four oriented views, (**b**_**5**_), (**b**_**6**_) connecting rod in two isometric views.

 Fig. 20 shows the checkerboard textured surface for vase and connecting rod, respectively. Similar with the double torus case shown in Fig. 13, there are two octagons on the textured surface of vase and connecting rod, too. It can be seen from Fig. 20(b_1_)–(b_6_) that two octagons with two colors sit next to each other for the case of connecting rod.

**Fig. 21:**
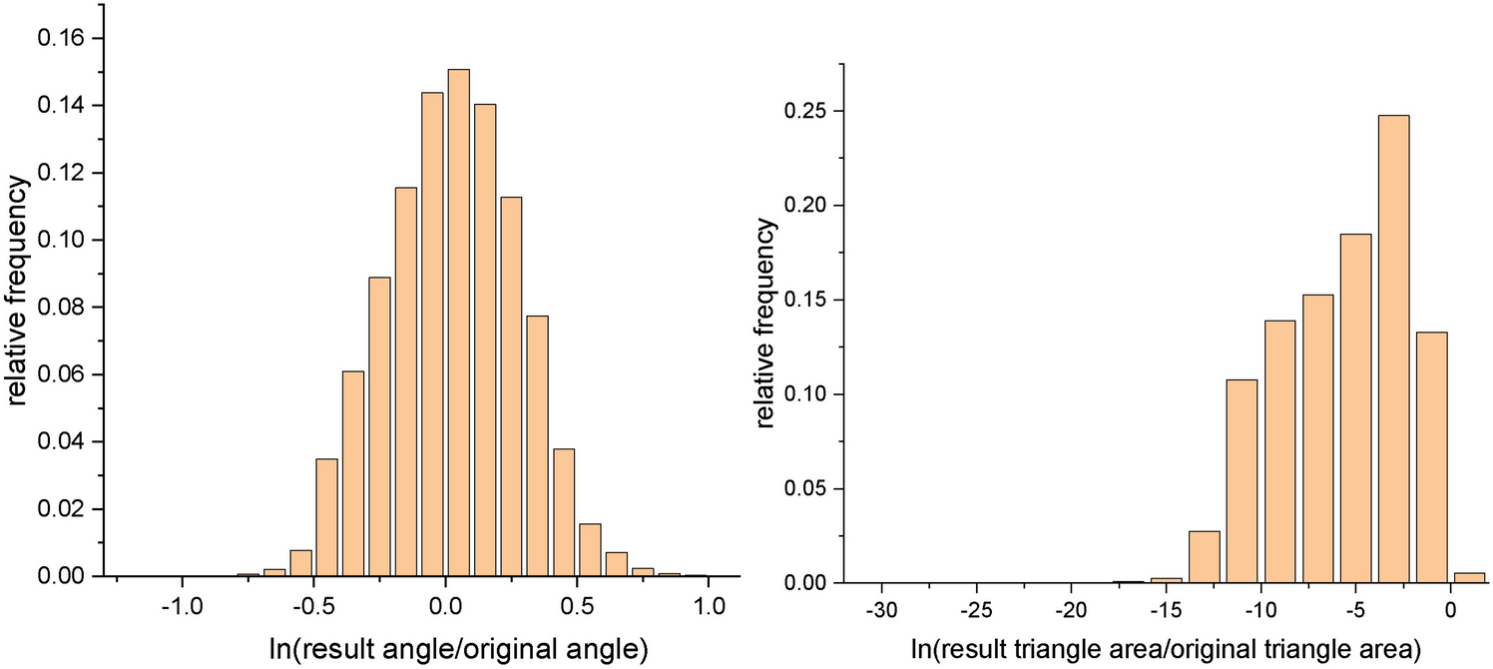
Histograms of angle distortion and area distortion for vase. Angle distortion (left), area distortion (right).

**Fig. 22:**
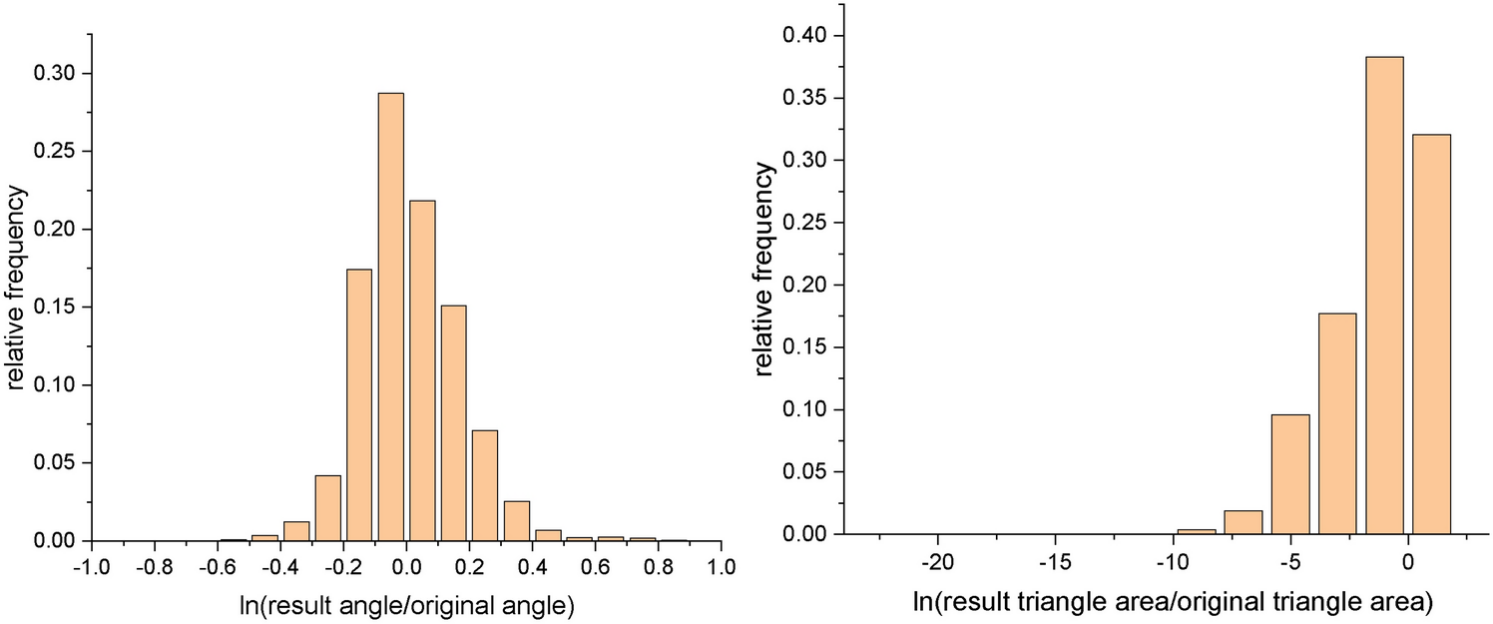
Histograms of angle distortion and area distortion for connecting rod. Angle distortion (left), area distortion (right).

Following the definition of angle distortion^28^, Figs. 21 and 22 show histograms of angle distortion and area distortion for vase and connecting rod, respectively. For each triangle in the mesh, we calculate the angles before and after the conformal mapping. And angle distortion measures how much the angles are distorted under a conformal map. The peaks shown in Figs. 21a and 22a are not as steep as anticipated. The reason may lie in the following aspects. Although conformal map in the smooth setting preserves angle, it will not strictly preserve angle in the discrete setting. That is to say, discrete conformal maps approximate conformal maps depending on whether the triangulation is fine enough or not^29^. Furthermore, in the discrete setting, if one tries to conformally map a surface to the plane while explicitly prescribing the boundary curve, the conformal distortion will be much larger that if one allows the boundary to evolve freely^30^.

### Surfaces of genus 3

#### Pushing forward the triangular meshes to the parameter domain for genus 3

**Fig. 23:**
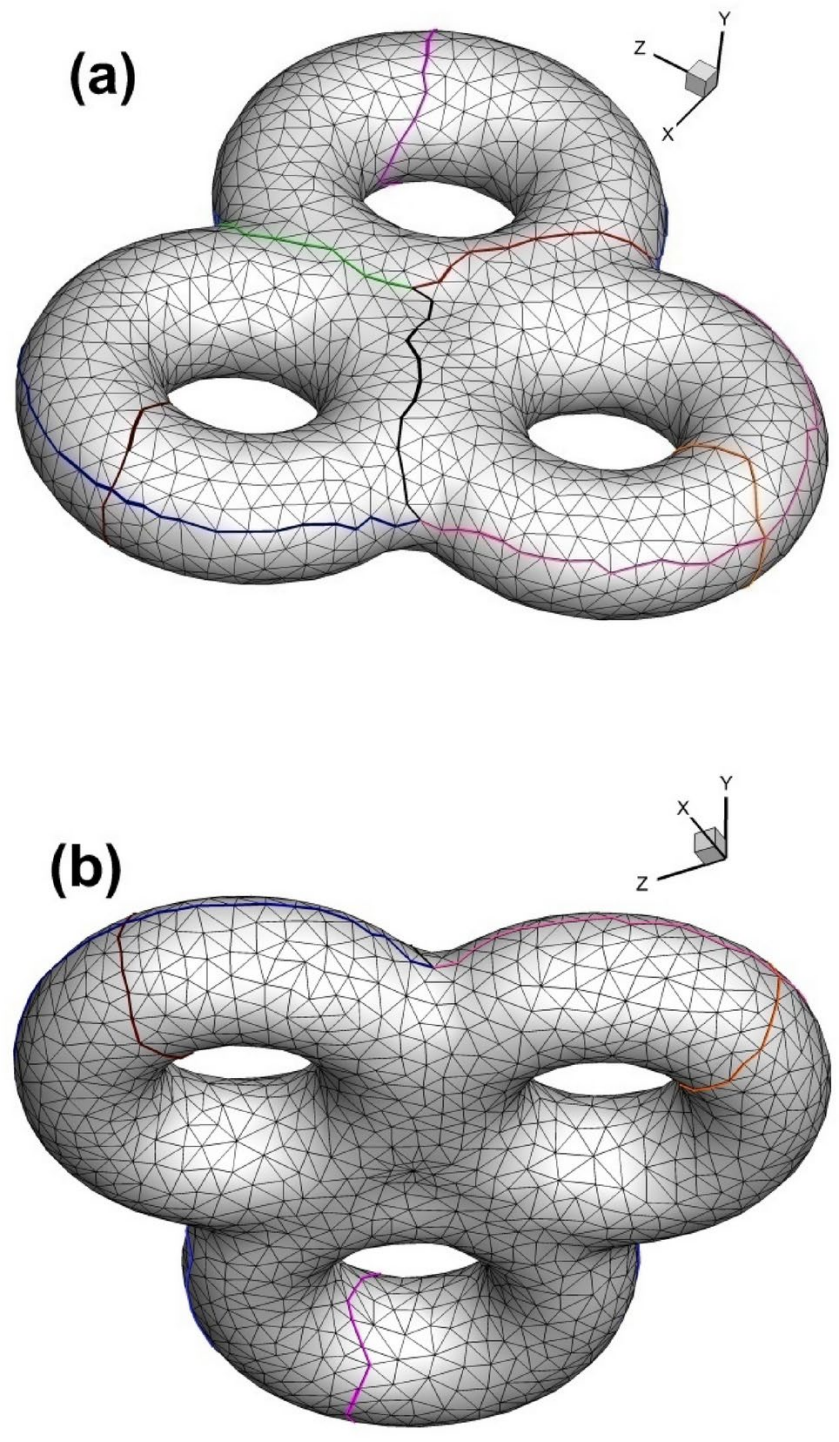
Two views of the triangular mesh of surface of genus 3 with cut graph. (**a**) Viewing from upper inclined side; (**b**) viewing from lower inclined side.

**Fig. 24:**
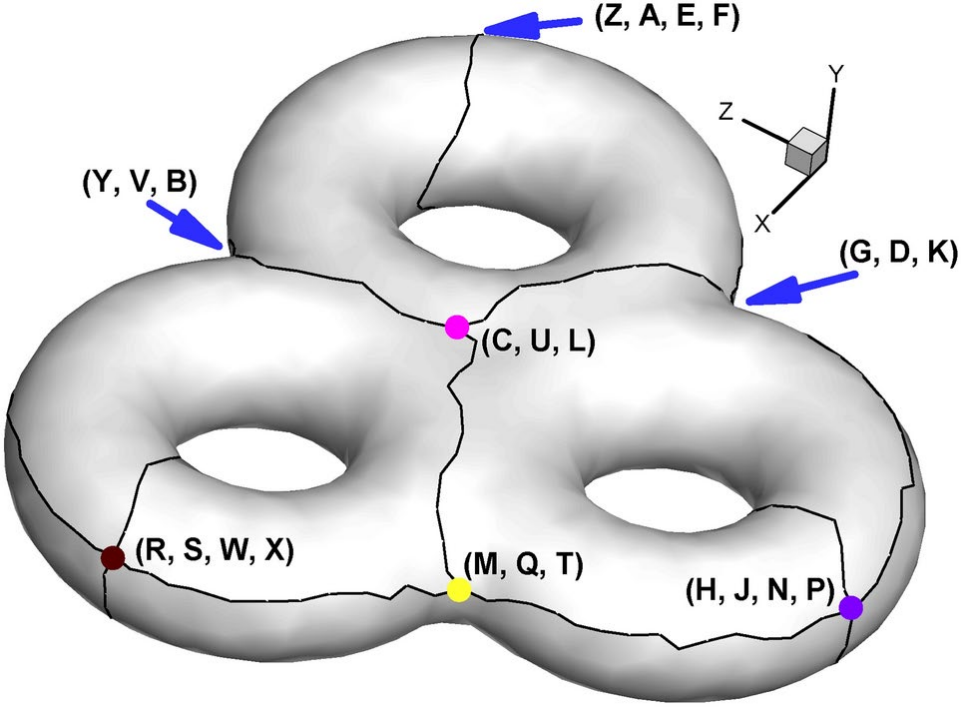
Illustration of 3-way branching vertex and 4-way branching vertex. There are four 3-way branching vertices and three 4-way branching vertices on the surface. 3-way branching vertex: (M, Q, T), (C, U, L), (Y, V, B), (G, D, K). 4-way branching vertex: (R, S, W, X), (Z, A, E, F), (H, J, N, P).

Fig. 23 shows the sketched handle loops and connection lines prior to the construction of the Euclidean Ricci flow. And Fig. 24 illustrates the 3-way branching vertex and 4-way branching vertex.

**Fig. 25:**
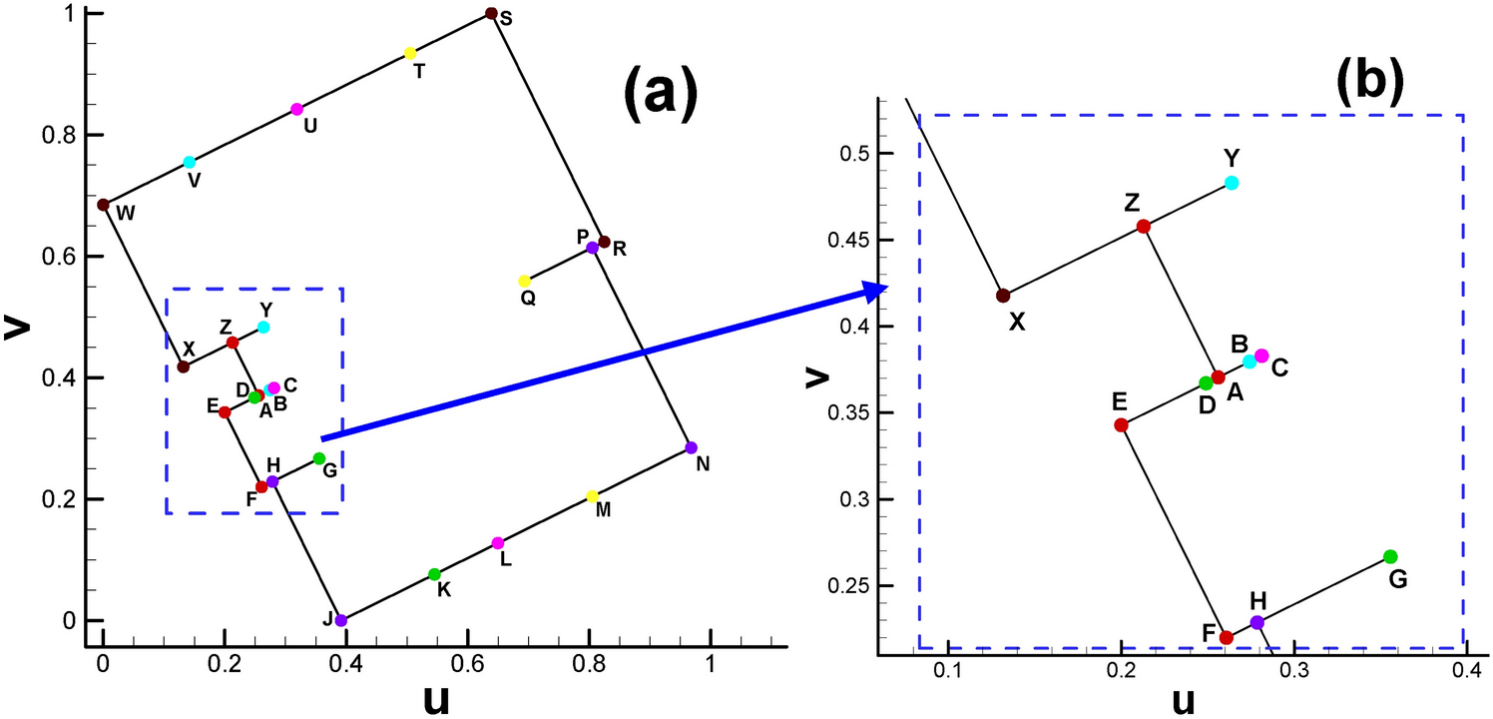
Illustration of child vertices in the parameter domain for genus 3. (**a**) Overall view; (**b**) close-up view. Child vertices of 3-way branching vertex: V, Y, B (cyan), C, U, L (purple), D, G, K (green), M, Q, T (yellow); child vertices of 4-way branching vertex: W, X, S, R (brown), Z, A, E, F(red), H, J, N, P (royal blue).

**Fig. 26:**
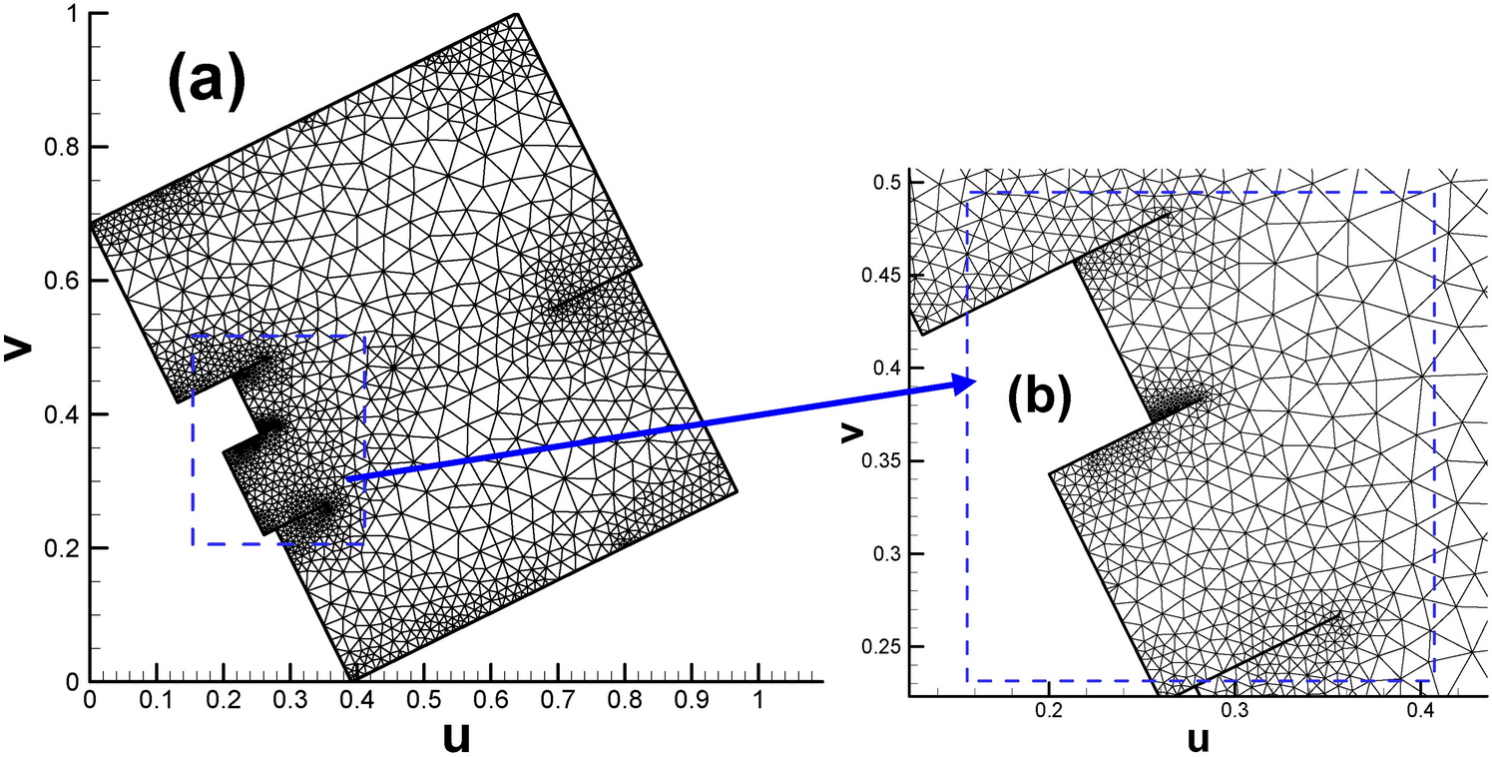
Embedding of triangular mesh of genus 3 surface in the parameter domain. (**a**) Overall view; (**b**) close-up view.

Fig. 25 and Table 4 show the positions of the child vertices of the 3-way branching vertex and 4-way branching vertex in the parameter domain. Similar to subsection  “Pushing forward the triangular meshes to the parameter domain for genus 3”, after being isometrically embedded in ${\mathbb{E}}^{2}$, the parameter domain of the surface of genus 3 takes the form of multiple connected rectangles, as shown in Fig. 26a, with Fig. 26b giving us insight into the meshes near coincidence lines.

**Table 4:** Major control parameters and partial calculation results of genus 3.

Vertex	Vertex number	Gaussian curvature	(u, v)	Vertex	Vertex number	Gaussian curvature	(u, v)
A	215	π/2	(0.2560, 0.3705)	N	1587	π/2	(0.9678, 0.2844)
B	1207	0	(0.2742, 0.3795)	P	1590	π/2	(0.8053, 0.6139)
C	1294	-π	(0.2812, 0.3829)	Q	1570	-π	(0.6936, 0.5588)
D	1615	0	(0.2490, 0.3670)	R	750	π/2	(0.8248, 0.6236)
E	214	π/2	(0.2000, 0.3429)	S	749	π/2	(0.6390, 1)
F	213	π/2	(0.2607, 0.2199)	T	1568	0	(0.5054, 0.9341)
G	1613	-π	(0.3556, 0.2667)	U	1293	0	(0.3189, 0.8420)
H	1589	π/2	(0.2786, 0.2287)	V	1205	0	(0.1418, 0.7546)
J	1588	π/2	(0.3914, 0)	W	748	π/2	(0, 0.6846)
K	1614	0	(0.5453, 0.07595)	X	747	π/2	(0.1317, 0.4177)
L	1295	0	(0.6497, 0.1274)	Y	1206	-π	(0.2638, 0.4829)
M	1569	0	(0.8054, 0.2043)	Z	212	π/2	(0.2129, 0.4578)

#### Design of the checkerboard texture in the parameter domain

**Fig. 27:**
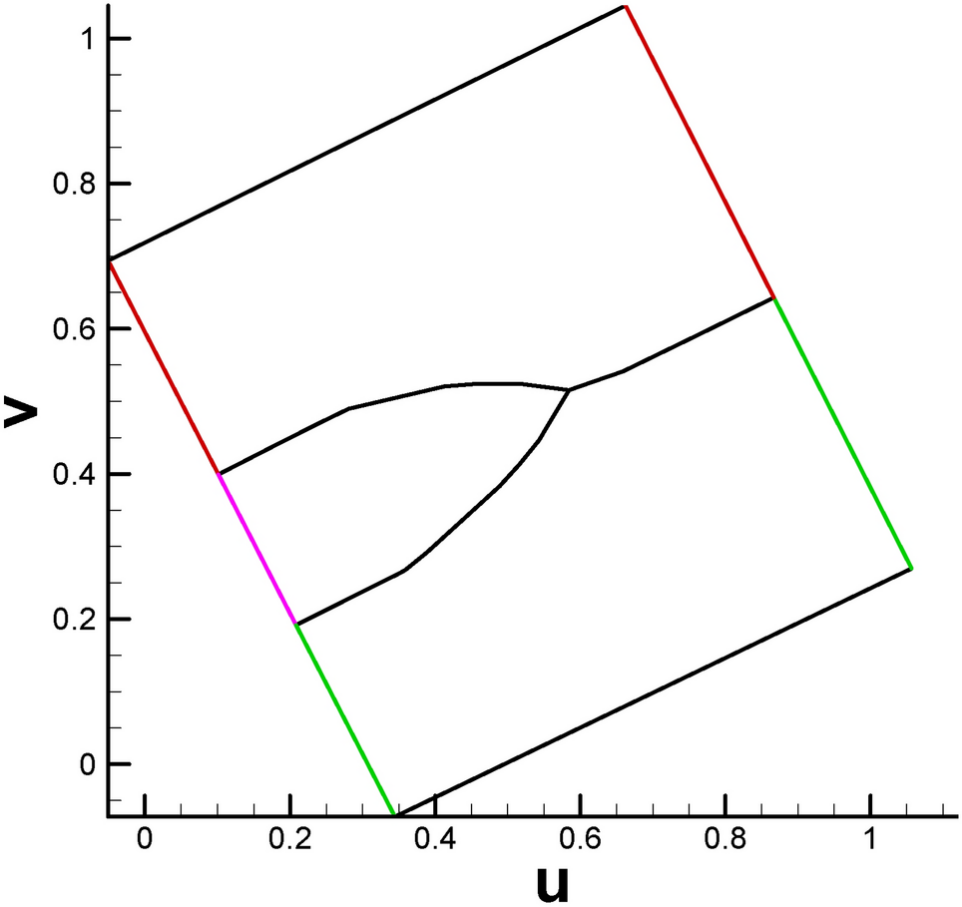
Initial division plan of parameter domain, with the same color lines representing paired lines.

Recalling the spatial position relationship between the pair of brother vertices in the genus 2 case, we can see that the two brother vertices (see the pair of (S_1_, S_2_) or (T_1_, T_2_) in Fig. 2) will always sit on two opposite lines (or called paired lines). However, the situation is more complicated for genus 3. Under this circumstance, there are two types of spatial position relationships between brother vertices. One type is similar to that in the genus 2 case; see the pair of (W, S) or (H, P) in Fig. 25a, namely, the child vertices are located in paired lines. For the other type, for the vertex on line ZA, its brother vertex will be on line FE, as shown in Fig. 25b. That is, there are no ordinary paired lines for this vertex. The inconsistent spatial position relationship of the brother vertex pair will lead to nontrivial isoparametric curves in the parameter domain. Analogous to streamlines in fluid dynamics, which are smooth and elegant, we provide an initial division plan for the parameter domain, as shown in Fig. 27.

**Fig. 28:**
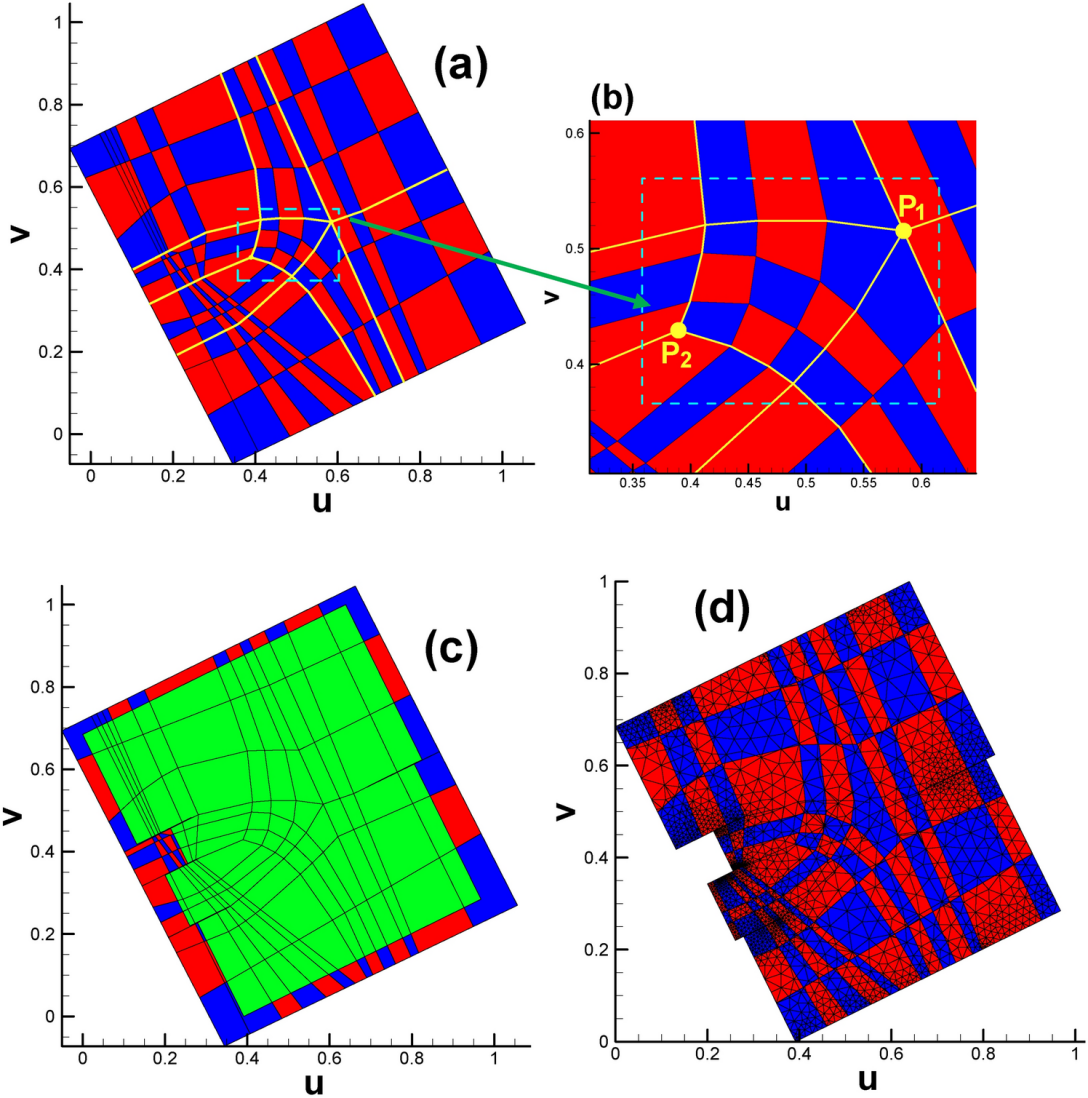
Subdividing triangular mesh with checkerboard texture (genus 3). (**a**) Designed checkerboard texture, with the vivid yellow curves sketching the division line; (**b**) close-up view of the P_1_ and P_2_ points, which are valence-3 and valence-5 points, respectively; (**c**) parameter domain with designed checkerboard texture; (**d**) triangular mesh in the parameter domain after being intersected with a slightly larger checkerboard texture zone.

Based on the division plan, we give the texture design (shown in Fig. 28a), which is generally slightly larger than the parameter domain shown in Fig. 25. When the larger texture domain is intersected with the embedded triangular mesh parameter domain (see Fig. 28b), we arrive at the designed checkerboard texture in ${\mathbb{E}}^{2}$ (see Fig. 28c).

By adopting the practice of using the topological valence^31^ of a vertex to discriminate a singularity vertex from a regular vertex, we can calculate the Gaussian curvatures of P_1_ and P_2_ in Fig. 28b, which are − π/2 and π/2, respectively. Therefore, the sum of their Gaussian curvatures is zero. For regular vertex, the Gaussian curvature is zero^31^.

**Fig. 29:**
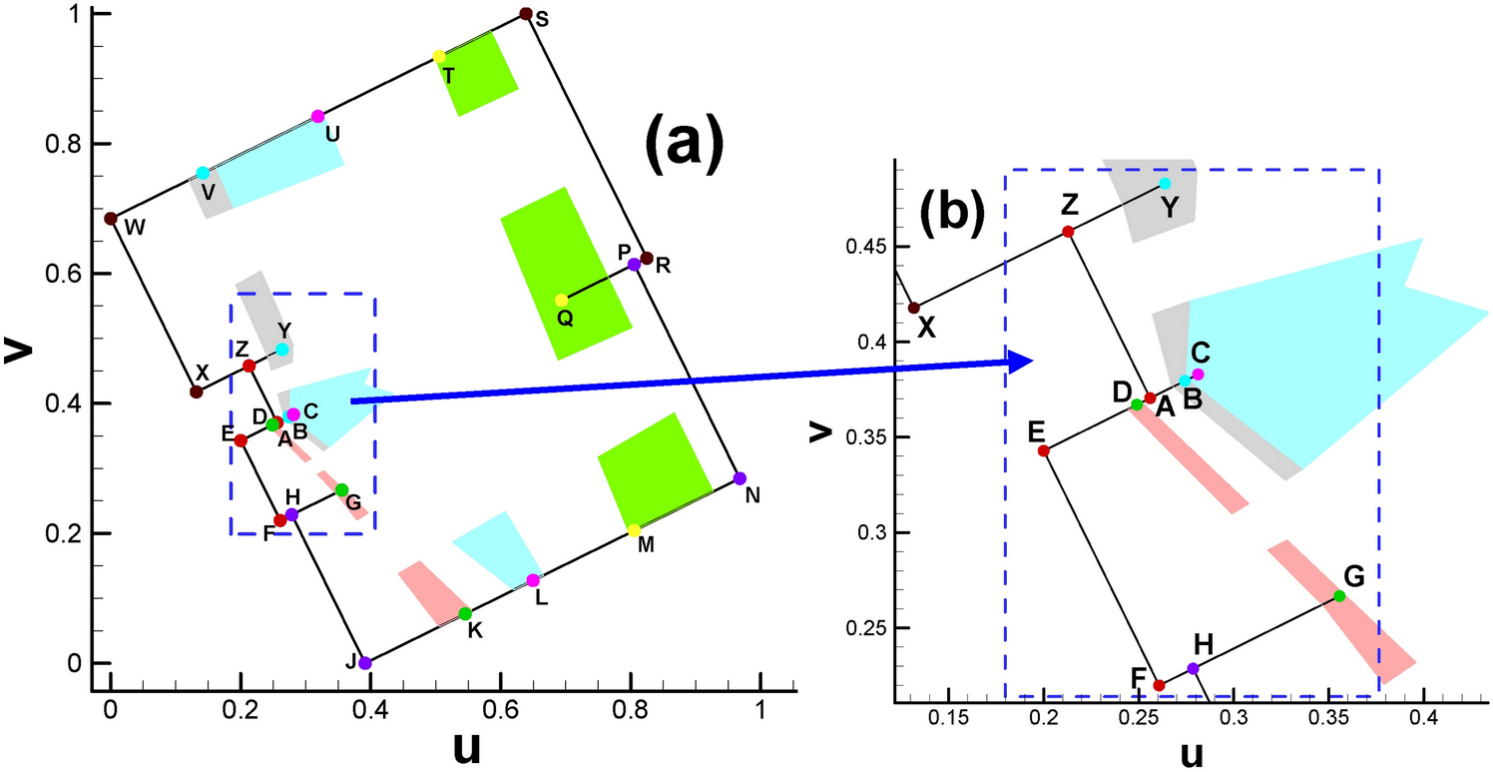
Illustration of 3-way branching vertex’s child vertices and their surrounding quadrilateral cells. (**a**) Overall view; (**b**) close-up view of the zone near coincidence line.

Fig. 28d shows the triangular mesh in the parameter domain after being intersected with a slightly larger checkerboard texture zone, illustrated in Fig. 28c. To better illustrate the position relationship between the child vertices of the 3-way branching vertex and its covering quadrilateral cell, we extract the corresponding data from Figs. 28d and 25a to display them in one figure. As shown in Fig. 29, every child vertex is surrounded by one quadrilateral cell. Therefore, a total of 3 child vertices are covered by three disjoint quadrilateral cells of the same color. There are four 3-way branching vertices on the original surface, leading to 12 child vertices and 12 different quadrilateral cells.

**Fig. 30:**
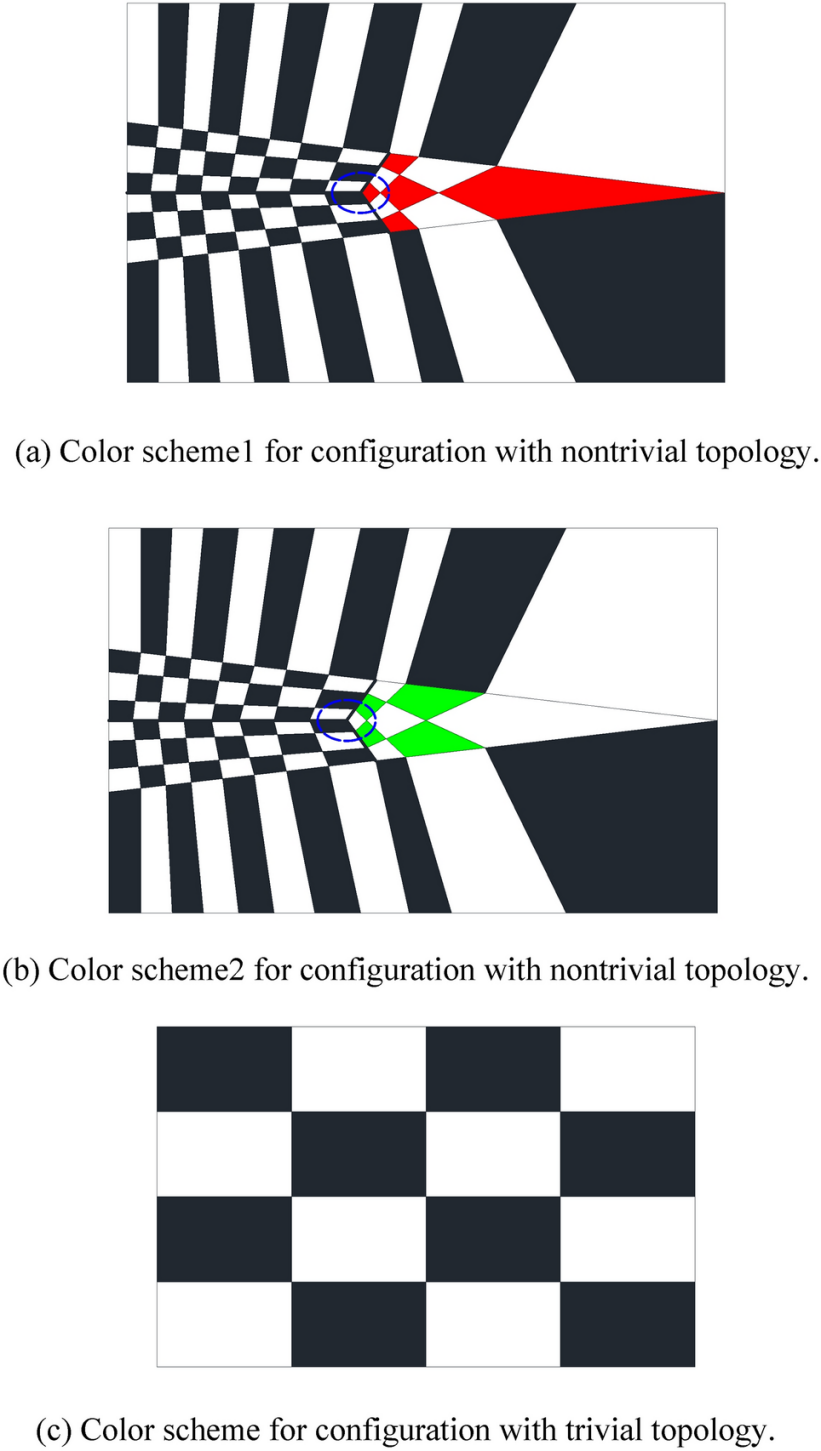
Illustration of color schemes for configuration with nontrivial and trivial topology.

Table 5 shows the detailed information of child vertices shown in Fig. 29. In addition, some quadrilateral cells with the same color sit together near points P_1_ and P_2_. We will briefly provide an explanation. Similar phenomena occur in Fig. 30a and b, where the rectangular zone has been completely divided into quadrilateral cells. However, regardless of which color scheme we choose, red or green, we cannot guarantee that the two colors are absolutely interlaced with each other. There are two quadrilateral cells with the same color deemed to sit together (see the cells circled by blue dashed line in Fig. 30a and b). The reason is that the configuration of Fig. 30a and b has a nontrivial topology instead of the easy circumstance in Fig. 30c.

**Table 5:** Detailed information of child vertices shown in Fig. 29.

Name of child vertex	Color of child vertex	Color of its covering quadrilateral cell	Its father vertex	Location of its father vertex
M	Yellow	Olive green	(M, Q, T) in Fig. 24	Octagon4 in Fig. 31
Q	Yellow	Olive green	(M, Q, T) in Fig. 24	Octagon4 in Fig. 31
T	Yellow	Olive green	(M, Q, T) in Fig. 24	Octagon4 in Fig. 31
C	Pink	Pale cerulean	(C, U, L) in Fig. 24	Octagon2 in Fig. 31
U	Pink	Pale cerulean	(C, U, L) in Fig. 24	Octagon2 in Fig. 31
L	Pink	Pale cerulean	(C, U, L) in Fig. 24	Octagon2 in Fig. 31
Y	Cyan	Gray	(Y, V, B) in Fig. 24	Octagon3 in Fig. 31
V	Cyan	Gray	(Y, V, B) in Fig. 24	Octagon3 in Fig. 31
B	Cyan	Gray	(Y, V, B) in Fig. 24	Octagon3 in Fig. 31
G	Green	Pink	(G, D, K) in Fig. 24	Octagon1 in Fig. 31
D	Green	Pink	(G, D, K) in Fig. 24	Octagon1 in Fig. 31
K	Green	Pink	(G, D, K) in Fig. 24	Octagon1 in Fig. 31

#### Generating textured surface for genus 3

**Fig. 31:**
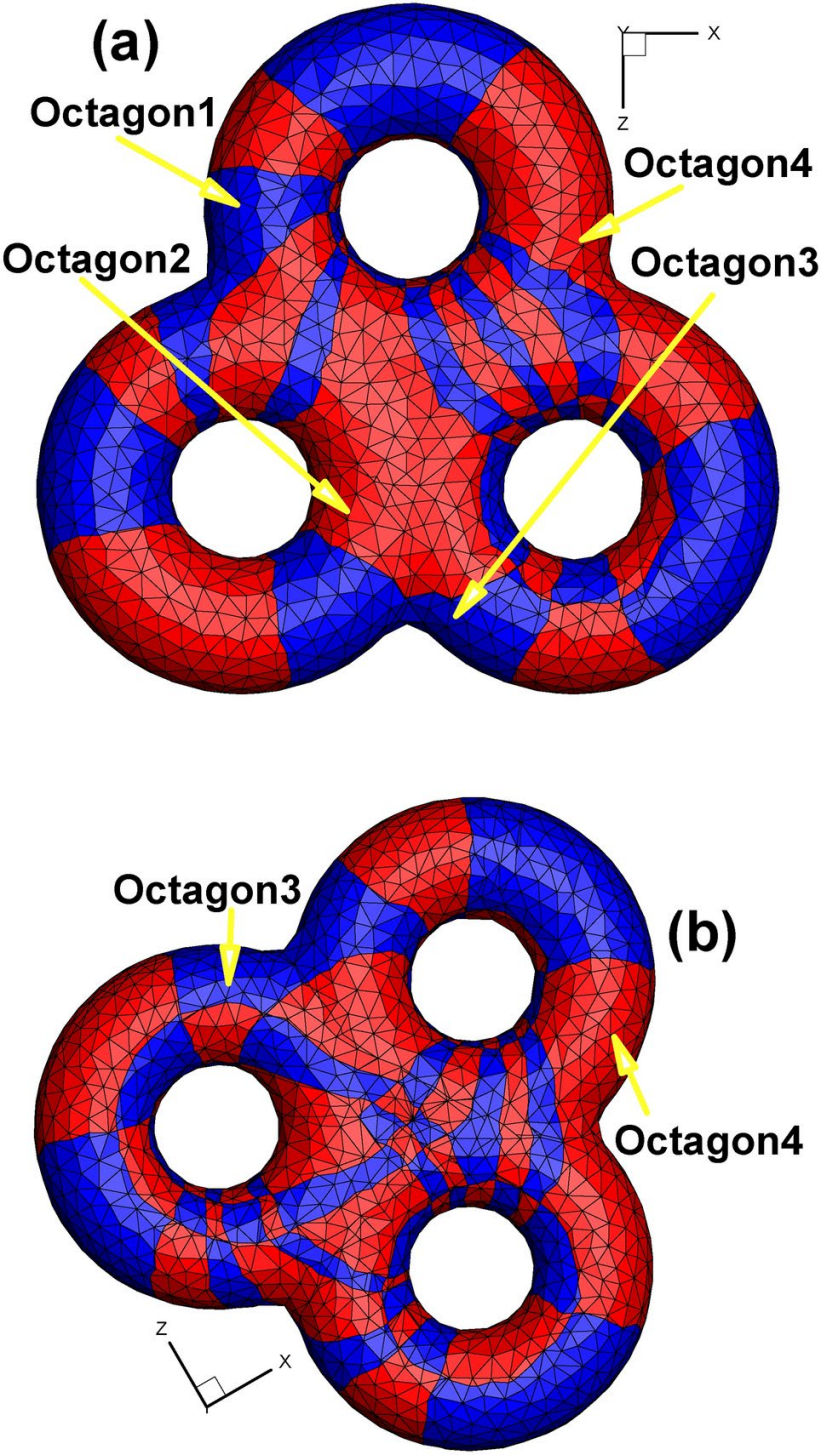
Checkerboard textured surface obtained by the Euclidean Ricci flow (genus 3). (**a**) Bottom view; (**b**) top view.

Fig. 31 shows the four octagons induced by four 3-way branching vertices. Based on the theory of the quad-mesh metric^31^, we can calculate the Gaussian curvature of each octagon as −2π. Since we have four octagons, their total Gaussian curvature is −8π, in good accordance with the regulation of the discrete Gauss-Bonnet theorem for the genus 3 surface.

## Conclusion and discussion

### Summary

It is still challenging to parameterize a high genus surface for both academic and industrial fields, which calls for wider attention from scientists and engineers. Based on traditional Euclidean Ricci flow, we devised a brief modification to the traditional type, which could only address the surface of genus 1; therefore, we propose a new but more powerful framework to parameterize high genus surfaces. By implementing our theory and algorithm of Euclidean Ricci flow, we calculate four examples to provide a vivid illustration of the parameterization method for high genus surfaces. Since our paper is very likely to be the first to use the Euclidean metric to parameterize high genus surfaces, we dispensed with the dogma and challenged the traditional view that only the hyperbolic Ricci flow can be used to parameterize high genus surfaces, thus broadening the horizon of application scenarios of the Euclidean Ricci flow. On the other hand, our method outperforms the hyperbolic Ricci flow in terms of its ability to show the spatial position of the singularity point in the parameter domain. Furthermore, compared with the hyperbolic Ricci flow, our method is more suitable for exploring the nature of singularity points on high genus surfaces, based on which it is easy to construct textures or quadrilateral meshes in the parameter domain. Therefore, our work contributes a missing piece to the Ricci flow framework, serving as a complementary augmentation to the already established hyperbolic Ricci flow framework.

### Limitations

There are still some limitations in the present study. Although the fundamental domain of the original surface obtained by our method can be embedded in ${\mathbb{E}}^{2}$, it cannot be tessellated. That is, ${\mathbb{E}}^{2}$ could not be covered by the fundamental domain of the original surface under any transformation group. Therefore, we must develop periodic boundary conditions, which are frequently used in computational fluid dynamics, to make textures smooth and continuous near the cut graph of the original surface.

This limitation is currently difficult to overcome because the situation is strongly determined by the nature of our method. However, since our work was mainly carried out on the original surface instead of its universal covering space^8^, the influence of the aforementioned limitations is relatively limited.

### Future directions

Since the cut graph adopted in this paper is manually sketched along different vertices, introducing the Birkhoff curve shortening method^32^ or the method to flip edges^33^ to generate geodesic paths will very likely shorten the length of the cut graph and hence make the textured surface smoother.

On the other hand, the method developed in this paper can be naturally extended to higher genus configurations.

For the time being, we simply adopt piecewise linear curves to construct the texture within the scope of the parameter domain, and we hope to achieve that in a more elegant way in the near future. Our paper is only the beginning of this research, and we welcome and encourage interested readers to join us and obtain in-depth results.

## Supplementary Information

Supplementary Information.

## Data Availability

All data generated or analysed during this study are included in this published article and its supplementary information files.
